# Dynamic Modulus Prediction of Fiber-Reinforced Asphalt Mixtures Based on XGBoost Optimized by an Improved Black-Winged Kite Algorithm

**DOI:** 10.3390/ma19173681

**Published:** 2026-08-29

**Authors:** Xunqian Xu, Shuyong Pan, Cheng Zhou, Wenxuan Ge, Xu Wu

**Affiliations:** 1 School of Transportation and Civil Engineering, Nantong University, Nantong 226019, China; xunqian_xu@ntu.edu.cn (X.X.); ntu_panshuyong@163.com (S.P.); zhou15283958510@163.com (C.Z.); 2 Department of AIOps & IoT Research, Nantong Institue of Technology, Nantong 226002, China; wuxu7109@163.com

**Keywords:** fiber-reinforced asphalt mixture, dynamic modulus, improved black-winged kite algorithm, XGBoost, interpretability analysis

## Abstract

Dynamic modulus is a key stiffness parameter in the mechanistic–empirical design of asphalt pavements. Traditional laboratory tests are time-consuming and costly, while conventional empirical models fail to characterize the nonlinear viscoelasticity introduced by fibers, and existing machine learning methods suffer from premature hyperparameter convergence and limited interpretability. To address these issues, this study employs an improved black-winged kite algorithm (IBKA) to optimize eXtreme Gradient Boosting (XGBoost) for establishing a dynamic modulus prediction model. Gaussian chaotic mapping, guided pool strategy, and adaptive step size are introduced to enhance global hyperparameter optimization capability. A dataset of 288 samples involving temperature, frequency, strain, and fiber categories is compiled from multi-condition tests. Nested cross-validation and an independent test set are adopted for internal optimization and generalization assessment, with permutation testing (1000 Monte Carlo, *p* < 0.001) confirming the statistical reliability of the model. The results demonstrate that IBKA–XGBoost delivers excellent accuracy and robustness, achieving an RMSE of 355.1248 MPa and an R^2^ of 0.9966 in NCV and 373.5450 MPa and 0.9955 on the independent test set. It outperforms BKA–XGBoost, four metaheuristic algorithms, and three conventional tuning strategies across nine evaluation metrics; compared with BKA–XGBoost, RMSE decreases by 23.9% and prediction uncertainty U_95_ narrows by 23.7%. SHAP and PDP analyses identify temperature as the dominant factor, reveal fiber-type differentiation governed by modulus matching and interfacial compatibility, and confirm asymmetric temperature–frequency interactions consistent with the time–temperature superposition principle. The proposed framework facilitates fiber screening and the intelligent refined design of pavement materials.

## 1. Introduction

Under the coupled conditions of increasing heavy-duty traffic and frequent extreme high- and low-temperature climates, conventional asphalt pavements commonly suffer from early distresses including rutting, cracking, and fatigue failure. Fiber-reinforced asphalt mixtures, with excellent crack-resistance reinforcement, deformation resistance and anti-fatigue performance, have become one of the important modifying materials for the performance improvement of high-grade highway pavements [1,2,3]. Dynamic modulus (DM) is the primary indicator that reflects the viscoelastic mechanical characteristics of fiber-reinforced asphalt mixtures and also acts as an essential input for structural response calculation, performance prediction and service life assessment in the mechanistic–empirical pavement design system. The precision of its acquisition directly governs the rationality and dependability of pavement design [4,5]. At present, DM is mainly obtained through standardized laboratory tests, which are inherently limited by high costs and long testing cycles. Therefore, constructing a high-precision DM prediction model for fiber-reinforced asphalt mixtures has become an urgent scientific and engineering task in road engineering.

To address the demand for predicting the DM of asphalt mixtures, a large number of researchers have carried out comprehensive and systematic investigations. Early studies mainly focused on empirical regression and semi-theoretical, semi-empirical models. Among them, the Witczak [6,7], Hirsch [8] and Al-Khateeb [9] models are the most extensively used and have been included in the pavement design codes of many countries. The Witczak model, developed on the basis of conventional hot-mix asphalt test data, establishes a correlation between DM and aggregate gradation, asphalt binder properties, and volumetric parameters via multivariate nonlinear regression, and it is recommended as the core model for MEPDG Level 3 design. The Hirsch and Al-Khateeb models are derived from the composite material mixing rule, considering the series–parallel combination and parallel arrangement of constituent phases in asphalt mixtures, respectively, thus possessing clearer physical significance. Although these models are simple in form, convenient in calculation, and stable in the prediction of conventional asphalt mixtures, their inherent drawbacks are significantly magnified when applied to fiber-modified asphalt mixtures. On the one hand, these models ignore critical factors such as fiber reinforcement, fiber–asphalt interfacial interaction and fiber distribution, so their applicability to fiber-modified systems is subject to certain limitations. On the other hand, these models rely on linear or quasilinear assumptions and cannot describe the strong nonlinear viscoelastic behavior caused by fiber addition [10]. In general, most traditional empirical/semi-empirical models are built on linear or quasilinear hypotheses, which results in considerable prediction errors in complex multiphase systems like fiber-reinforced asphalt mixtures, which can hardly satisfy the precision requirements for the refined design of long-life pavements.

With the rapid advancement of machine learning techniques, algorithms including Artificial Neural Network (ANN), Support Vector Regression (SVR), Random Forest (RF), and eXtreme Gradient Boosting (XGBoost) have been gradually applied to the DM prediction of asphalt mixtures, achieving substantially better performance than traditional empirical models [11,12,13,14,15,16,17,18,19,20,21,22,23,24]. Among them, XGBoost, grounded in a regularized gradient boosting architecture, exhibits superior advantages in nonlinear fitting, anti-interference capability, and generalization, which make it a preferred model in the performance prediction of asphalt mixtures [5,11,25,26]. Nevertheless, three critical technical gaps remain in existing studies on DM prediction. First, most models concentrate on conventional or recycled asphalt mixtures, with limited systematic research on fiber-modified asphalt mixtures; moreover, fiber type is rarely included as a key input feature, which fails to reflect the differential regulation of various fibers on DM and, thus, limits engineering applicability. Second, the prediction performance of XGBoost is greatly affected by hyperparameter combinations, while traditional tuning methods are inefficient and prone to local optima. Conventional metaheuristic algorithms such as Particle Swarm Optimization (PSO) [27] and Whale Optimization Algorithm (WOA) [28] still suffer from premature convergence and limited optimization accuracy when optimizing XGBoost, preventing full exploitation of the model’s potential. Third, most existing studies prioritize prediction accuracy, which leads to a prevalent “black-box” problem. The few interpretability analyses are limited to feature importance ranking, without systematically revealing the fiber influence laws or multi-factor coupling mechanisms, which hinders the support of refined material design and engineering applications.

Metaheuristic optimization methods offer a viable strategy for the global hyperparameter optimization of XGBoost. As one such method, the black-winged kite algorithm (BKA) is a recently developed swarm intelligence optimizer [29] that performs global search by mimicking the diving attack, circling hunting, and collective migration patterns of black-winged kites. Relative to conventional algorithms like PSO and WOA, BKA is characterized by a straightforward structure, few control parameters, and strong global exploration ability, showing promising prospects in continuous function optimization and structural engineering design [30,31,32]. However, the original BKA has inherent limitations including uneven population initialization, premature convergence, and fixed step-size adjustment, which leave room for improvement in high-dimensional hyperparameter optimization [33]. Therefore, it is necessary to improve BKA to better match the hyperparameter tuning requirements of XGBoost. Meanwhile, the combined framework of SHapley Additive exPlanations (SHAP) and Partial Dependence Plot (PDP) has been extensively and reliably validated in the performance prediction of advanced cement-based composites, various civil engineering structural materials, and traditional pavement materials [34,35,36,37,38,39,40]. This framework can effectively quantify feature contributions, reveal single-factor influence laws, and uncover multi-factor coupling mechanisms. However, its systematic application and in-depth mechanistic analysis remain scarce in the DM prediction of fiber-reinforced asphalt mixtures.

To address the above issues, this paper proposes an improved black-winged kite algorithm (IBKA). Gaussian chaotic mapping is adopted to improve the initial population distribution, a guidance pool strategy is introduced to maintain the diversity of search directions, and an adaptive step-size strategy is designed to achieve a smooth transition between exploration and exploitation. On this basis, the nine core hyperparameters of XGBoost are optimized to establish the IBKA–XGBoost model for dynamic modulus prediction. The model takes experimental temperature, loading frequency, strain level, and fiber type as input features and dynamic modulus as the output response. For model evaluation, nested cross-validation (NCV) is introduced to achieve mathematical separation between hyperparameter calibration and performance assessment. An independent test set is strictly isolated throughout the whole workflow; it is not involved in any model fitting or parameter calibration and is only used for final generalization evaluation. Furthermore, SHAP and PDP are combined for interpretability analysis to systematically reveal the influence rules and interaction mechanisms of various factors on dynamic modulus. This study aims to provide a data-driven theoretical basis and technical support for the rapid prediction of dynamic modulus, fiber selection, and the engineering design of fiber-reinforced asphalt mixtures.

## 2. Experimental Materials and Methods

### 2.1. Raw Materials and Specimen Preparation

To establish a standard dataset suitable for machine learning modeling, this study selected SAC-13 discontinuous-graded fiber-reinforced asphalt mixture. The gradation was constructed using the power function method, and the passing rates of key sieve sizes are shown in Table 1. Coarse aggregate (5–10 mm and 10–15 mm diabase crushed stone) was sourced from Nalian Yanxiangshan Quarry, Tiandong, Guangxi, China; fine aggregate (0–3 mm diabase manufactured sand) was sourced from Yongfu Luojin Quarry, Guangxi, China; and mineral filler (ground limestone) was produced by Liuzhou Tiancheng Quarry, Liuzhou, Guangxi, China. SBS-modified asphalt produced by Shell Company of the United States (Houston, TX, USA) was adopted as the asphalt binder. Five types of fibers widely used in engineering were selected, including polyethylene terephthalate fiber (PET1), polyester fiber (PET2), polyacrylonitrile fiber (PAN), polyvinyl alcohol fiber (PVA), and lignin fiber (CEL). A fiber-free control group was also set up, and the dosage of all fibers was uniformly 0.3%. This dosage was determined through preliminary single-factor tests, which can ensure the uniform dispersion of fibers and achieve both modification effect and construction workability.

Coarse aggregate (5–10 mm and 10–15 mm diabase crushed stone) was sourced from Nalian Yanxiangshan Quarry, Tiandong, Guangxi, China; fine aggregate (0–3 mm di-abase manufactured sand) was sourced from Yongfu Luojin Quarry, Guangxi, China; and mineral filler (ground limestone) was produced by Liuzhou Tiancheng Quarry, Liuzhou, Guangxi, China. SBS-modified asphalt (Shell Oil Company, Houston, TX, USA) was adopted as the asphalt binder. Five types of fibers widely used in engineer-ing were selected, including polyethylene terephthalate fiber (PET1) and polyester fiber (PET2) —provided by Nantong Xindike Monofilament Technology Co., Ltd., Nantong, Jiangsu, China—as well as polyacrylonitrile fiber (PAN) and polyvinyl alcohol fiber (PVA) (provided by Anhui Wanwei High-tech Materials Co., Ltd., Hefei, Anhui, China), and lignin fiber (CEL) —provided by Nantong Xindike Monofilament Technology Co., Ltd., Nantong, Jiangsu, China. A fiber-free control group was also set up, and the dosage of all fibers was uniformly 0.3%.

The Marshall design procedure was utilized to identify the optimum asphalt–aggregate ratio (OAC) for each mixture group. Five levels of asphalt–aggregate ratios were investigated, with stability, flow value, and air voids as the primary control criteria. The optimal asphalt–aggregate ratios for each mixture were identified as 5.2% for the control group, 5.5% for PET1, 5.1% for PET2, 5.4% for PAN, 5.4% for PVA, and 5.7% for CEL. All volumetric and mechanical properties of the fabricated specimens complied with the specification limits, thereby ensuring the stability and reliability of the subsequent DM test data. The physical and mechanical properties of the five types of fibers are listed in Table 2. PET1 exhibits the highest breaking elongation of 18.3% and a dry heat shrinkage rate of 9.5% among all tested fibers. PVA possesses an ultra-high elastic modulus of 39.71 GPa. PET2 delivers a tensile strength exceeding 500 MPa but presents a low breaking elongation of only 5.55%. PAN shows an ultra-fine fineness of 1.2 dtex, while CEL is a flocculent short fiber with a density of 1.54 g/cm^3^ and an average fiber length of 1.1 mm. The significant differences in the inherent physical and mechanical properties of various fibers provide a fundamental physical basis for analyzing their differentiated regulation effects on the dynamic modulus of asphalt mixtures.

### 2.2. Test Methods

#### 2.2.1. Dynamic Mechanical Property Test

**Figure 1:**
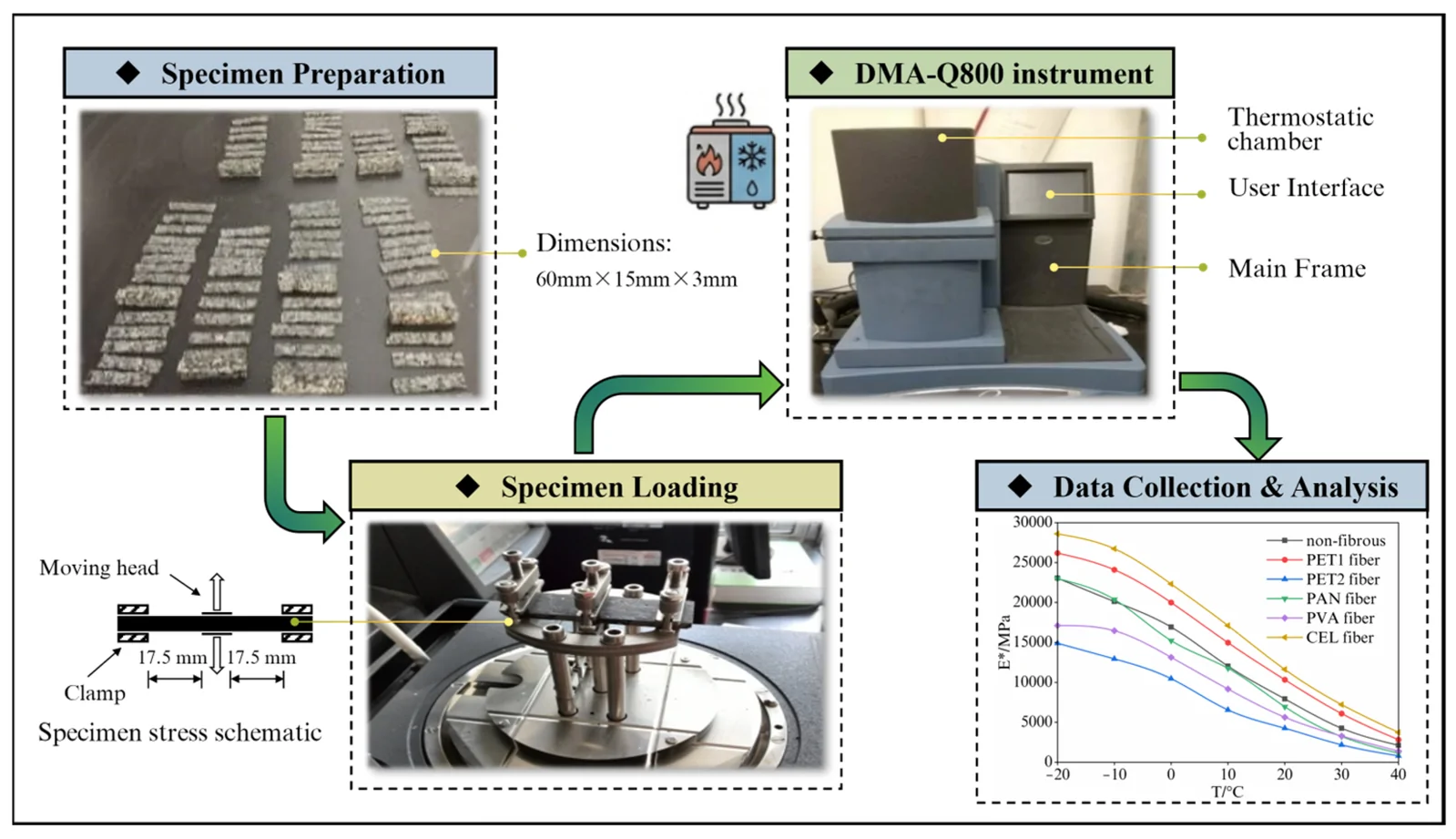
Test Flow.

The dynamic mechanical analysis (DMA) technique was utilized to evaluate the viscoelastic behavior and measure the DM of the fiber-modified asphalt mixtures, us-ing a Q800 dynamic mechanical analyzer (TA Instruments, New Castle, DE, USA). All test samples were prepared in prismatic form with dimensions of 60.0 mm × 15.0 mm × 3.0 mm, with rigorous quality control applied to ensure flat, crack-free surfaces, thus ensuring uniform stress distribution and reliable test outcomes. To reduce experimental uncertainty, four replicate samples were prepared under each test condition, with the arithmetic average of the measured values taken as the final reported result. The test setup was configured to simulate the real service environment of asphalt pavements, covering temperatures (−20 °C to 40 °C), loading frequencies (0.1–80 Hz), and strain levels (30–90 με). This comprehensive test matrix ensures the representativeness and generalization capability of the dataset for subsequent machine learning modeling. The detailed test workflow is presented in Figure 1.

#### 2.2.2. Data Collection and Preprocessing

A total of more than 300 raw datasets were collected in this test. After visual inspection and loading stability assessment, the Grubbs test was applied to eliminate outliers, yielding 288 valid datasets for subsequent modeling. For the categorical feature of fiber type, label encoding was performed to convert the qualitative attribute into a numerical form compatible with machine learning algorithms: no fiber = 0, PET1 = 1, PET2 = 2, PAN = 3, PVA = 4, and CEL = 5.

### 2.3. Data Analysis

#### 2.3.1. Descriptive Statistical Analysis

Descriptive statistical analysis was performed on the 288 valid datasets, with results listed in Table 3. In this study, experimental temperature, loading frequency, strain level, and fiber type were selected as input features, while DM was taken as the output response. All input variables exhibited wide ranges and uniform distributions, fully covering the in-service conditions of actual pavements. The coefficient of variation of the dataset was within a reasonable range, without significant skewness, which indicates satisfactory representativeness and diversity of the samples.

#### 2.3.2. Correlation Analysis

**Figure 2:**
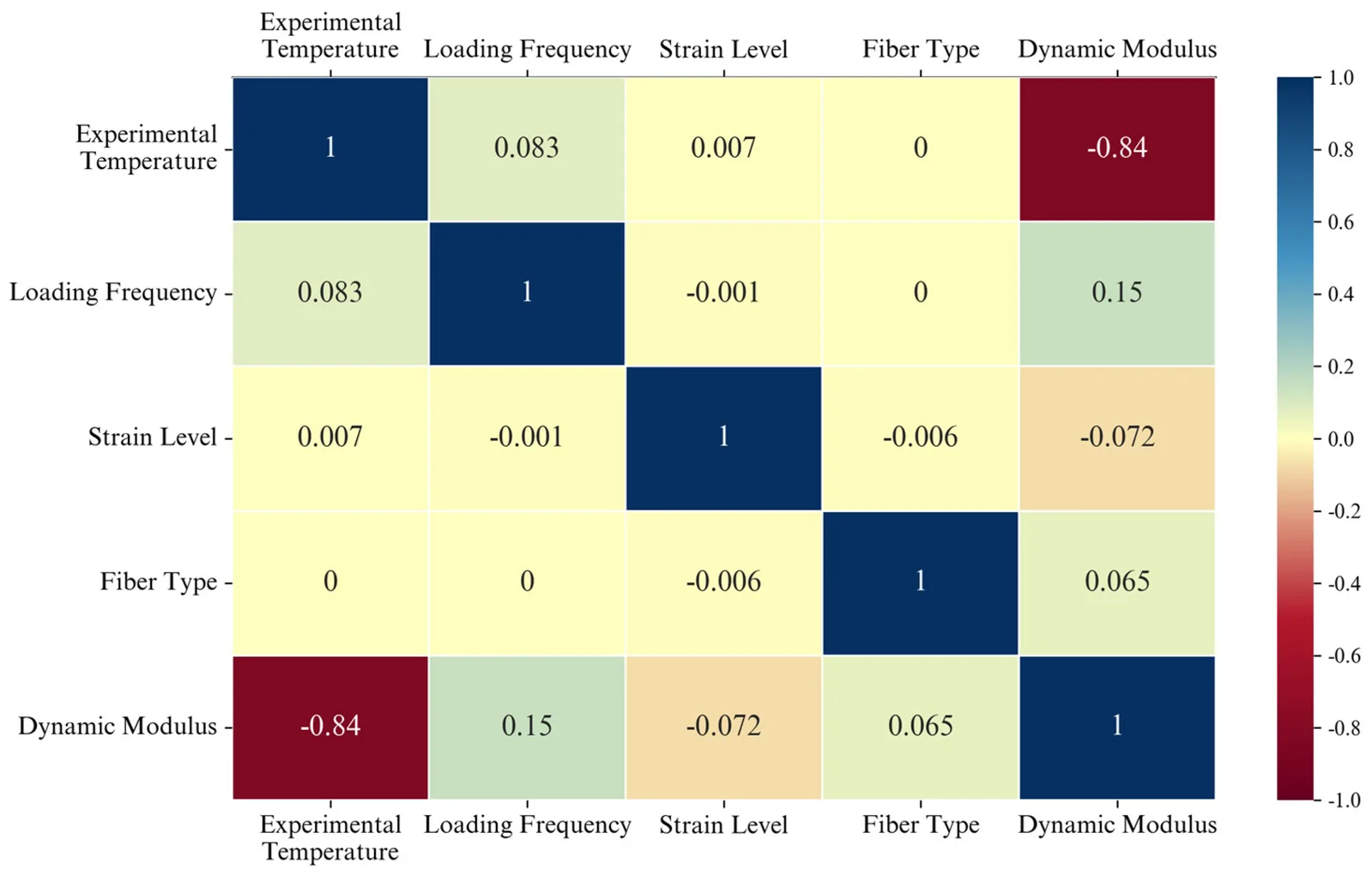
Pearson Correlation Coefficient Matrix.

A Pearson correlation matrix analysis was conducted to assess the interrelationships between the input features, with the outcomes visualized in Figure 2. The results showed that the absolute correlation coefficients between all pairs of input variables were below 0.1, with the highest value reaching only 0.083, far below the threshold for significant correlation (0.8) [41]. Multicollinearity may result in unstable model training and biased coefficient estimates. As no severe multicollinearity was identified in the dataset, dimensionality reduction methods like principal component analysis were considered unnecessary. Original features can be directly used for model training, which ensures the integrity of feature information and stability of modeling. These results confirm the rationality of using these variables to predict the DM of fiber-reinforced asphalt mixtures.

## 3. Development of the Prediction Model

This section systematically elaborates the mathematical principles and improvement mechanisms of eXtreme Gradient Boosting (XGBoost) and the improved black-winged kite algorithm (IBKA). The IBKA–XGBoost dynamic modulus (DM) prediction model is constructed, and the methods for model performance evaluation and interpretability analysis are specified, so as to provide theoretical and methodological support for subsequent prediction and mechanism analysis. All models were implemented in Python 3.9.13 using XGBoost 3.2.0, NumPy 2.4.2, Pandas 2.3.3, and Scikit-learn 1.8.0.

### 3.1. eXtreme Gradient Boosting (XGBoost)

XGBoost is a highly scalable gradient boosting framework introduced by Chen and Guestrin [42]. Thanks to its robust learning capacity and consistent generalization performance, it has found extensive application in diverse engineering prediction tasks [43,44,45]. Its mathematical expression is as follows:

(1) $$L=\sum_{i}\;l({\hat{y}}_{i},y_{i})+\sum_{j}\;\Omega (\theta_{j})$$

where l denotes the loss function between the predicted value ${\hat{y}}_{i}$ and the true value $y_{i}$, and $\Omega (\theta_{j})$ is the regularization term for the j-th regression tree, designed to control model complexity. The expression for $\Omega (\theta_{j})$ is given by:

(2) $$\Omega (\theta_{j})=\gamma T_{j}+\frac{1}{2}\lambda ∥w_{k}∥=\gamma T_{j}+\frac{1}{2}\lambda \sum_{k=1}^{T_{j}}\;{\left[w_{k}^{\left(j\right)}\right]}^{2}$$

where γ is the minimum loss reduction required for node splitting, $T_{j}$ is the number of leaf nodes in the j-th tree, λ is the regularization coefficient for leaf weights, and $w_{k}^{\left(j\right)}$ is the weight of the k-th leaf node in the regression tree.

### 3.2. Metaheuristic Optimization Algorithms

The predictive performance of machine learning models is highly contingent on appropriate hyperparameter configurations. Manual tuning is inefficient and prone to entrapment in local optima. Employing high-performance metaheuristic algorithms for global hyperparameter optimization can significantly improve computational efficiency and prediction accuracy.

#### 3.2.1. Black-Winged Kite Algorithm (BKA)

The BKA achieves global optimization by simulating the predatory attack and group migration behaviors of black-winged kites. The algorithm includes three core stages: population initialization, attack phase, and migration phase.

(1) Population initialization

The positions of the black-winged kite population are represented as a solution matrix in dom dimensions:

(3) $$X=\left[\begin{array}{cccc} X_{1,1} & X_{1,2} & \cdots & X_{1,dom}\\ X_{2,1} & X_{2,2} & \cdots & X_{2,dom}\\ \vdots & \vdots & & \vdots \\ X_{pop,1} & X_{pop,2} & \cdots & X_{pop,dom} \end{array}\right]$$

Initial positions are randomly generated within the search boundaries:

(4) $$X_{i}=X_{lb}+rand(X_{ub}-X_{lb})$$

where pop denotes the population size; $X_{ub}$ and $X_{lb}$ are the upper and lower limits of the search space, respectively; and rand is a uniform random number in [0, 1].

(2) Attack phase

The BKA approach preys through two modes: circling adjustment and global exploration. The position update rule is defined as:

(5) $$x_{t+1}^{i,j}=\left\{\begin{array}{l} x_{t}^{i,j}+n\left(1+sin(rand)\right)\times x_{t}^{i,j},p<r\\ x_{t}^{i,j}+n\times (2r-1)\times x_{t}^{i,j},else \end{array}\right.$$

where p is a constant set to 0.9 and n is the step factor that decreases nonlinearly:

(6) $$n=0.05\times e^{-2\times {\left(\frac{t}{T}\right)}^{2}}$$

where T is the maximum number of iterations.

(3) Migration phase

When the fitness of an individual is poor, BKA enters the migration stage to escape local optima via large-scale position updates. The position update rule is:

(7) $$x_{t+1}^{i,j}=\left\{\begin{array}{c} x_{t}^{i,j}+C(0,1)\times \left(x_{t}^{i,j}-L_{t}^{j}\right),F_{i}<F_{ri}\\ x_{t}^{i,j}+C(0,1)\times \left(L_{t}^{j}-m\times x_{t}^{i,j}\right),else \end{array}\right.$$

where $L_{t}^{j}$ is the dimensionwise leader, $F_{i}$ is the current fitness, $F_{ri}$ is the fitness of a random individual, C(0,1) follows standard Cauchy distribution, and m is calculated as:

(8) $$m=2\times sin\left(r+\frac{\pi }{2}\right)$$

#### 3.2.2. Improved Black-Winged Kite Algorithm (IBKA)

To address the drawbacks of the original BKA, including uneven population initialization, premature convergence, and fixed step-size adjustment, IBKA is developed by combining Gaussian chaotic mapping, a guided pooling strategy, and an adaptive step-size strategy.

(1) Gaussian chaotic mapping

Gaussian chaotic mapping [46] replaces random initialization to improve population diversity and uniformity. Its iterative formula is:

(9) $$\left.z_{n+1}=\left\{\begin{array}{ll} 0 & z_{n}=0\\ \frac{1}{z_{n}mod\left(1\right)}=\frac{1}{z_{n}}-\left[\frac{1}{z_{n}}\right] & z_{n}\neq 0 \end{array}\right.\right.$$

where $z_{n}$ is the chaotic value at the current iteration and $z_{n+1}$ is the chaotic value at the next iteration. The chaotic values are linearly mapped to the search boundaries of the algorithm, which yields the new population initialization formula:

(10) $$X_{i}=z_{ij}\cdot (X_{ub}-X_{lb})+X_{lb}$$

(2) Guided pooling strategy

A guided pool containing optimal and mean information is constructed to guide position updating and strengthen global search capability:

(11) $$X_{pool}=\left\{\begin{array}{c} X_{best1},X_{best2},X_{best3},X_{mean1},X_{mean2} \end{array}\right\}$$

A guide point is randomly selected to revise the attack-phase update rule:

(12) $$x_{t+1}^{i,j}=\left\{\begin{array}{ll} x_{t}^{i,j}+n\cdot (1+sin(rand))\cdot \left(X_{guide,j}-x_{t}^{i,j}\right) & p<r\\ X_{guide,j}\cdot \left(n\cdot \left(2r-1\right)+1\right) & else \end{array}\right.$$

(3) Adaptive step-size strategy

A sinusoidal adaptive step-size factor is designed to realize a smooth transition between large-scale global exploration in the early stage and small-scale fine exploitation in the later stage of iterations. The expression of the adaptive step size is:

(13) $$\left\{\begin{array}{l} step=1-\frac{t}{T}\\ amp=0.1+0.4\cdot sin(\pi \cdot step)\\ {ASS}_{step}=amp\cdot tan((rand(1,dim)-0.5)\pi ) \end{array}\right.$$

where step is the iterative attenuation factor, decreasing linearly from 1 to 0 with iterations, and amp is the sinusoidal adaptive amplitude, which realizes smooth step-size transition via a sine function to avoid abrupt oscillation, with a range of [0.1, 0.5].

The migration-phase update formula is improved accordingly:

(14) $$x_{t+1}^{i,j}=\left\{\begin{array}{ll} x_{t}^{i,j}+{ASS}_{step}\cdot (x_{t}^{i,j}-L_{t}^{j}) & F(X_{i})<F(X_{r})\\ x_{t}^{i,j}+{ASS}_{step}\cdot \left(L_{t}^{j}-m\cdot x_{t}^{i,j}\right) & else \end{array}\right.$$

#### 3.2.3. Performance Validation of the IBKA

To evaluate the performance of the proposed optimization approach, the CEC2022 benchmark functions were used to compare PSO, WOA, Dung Beetle Optimizer (DBO) [47], Coati Optimization Algorithm (COA) [48], the original BKA, and IBKA. To ensure fair comparison, all algorithms were run under the same parameter settings: population size = 30, maximum iterations = 500, and problem dimension = 10. Each test function was executed 30 times independently; the mean and standard deviation were recorded as key metrics, and the Friedman test was applied to rank the overall optimization capability.

**Figure 3:**
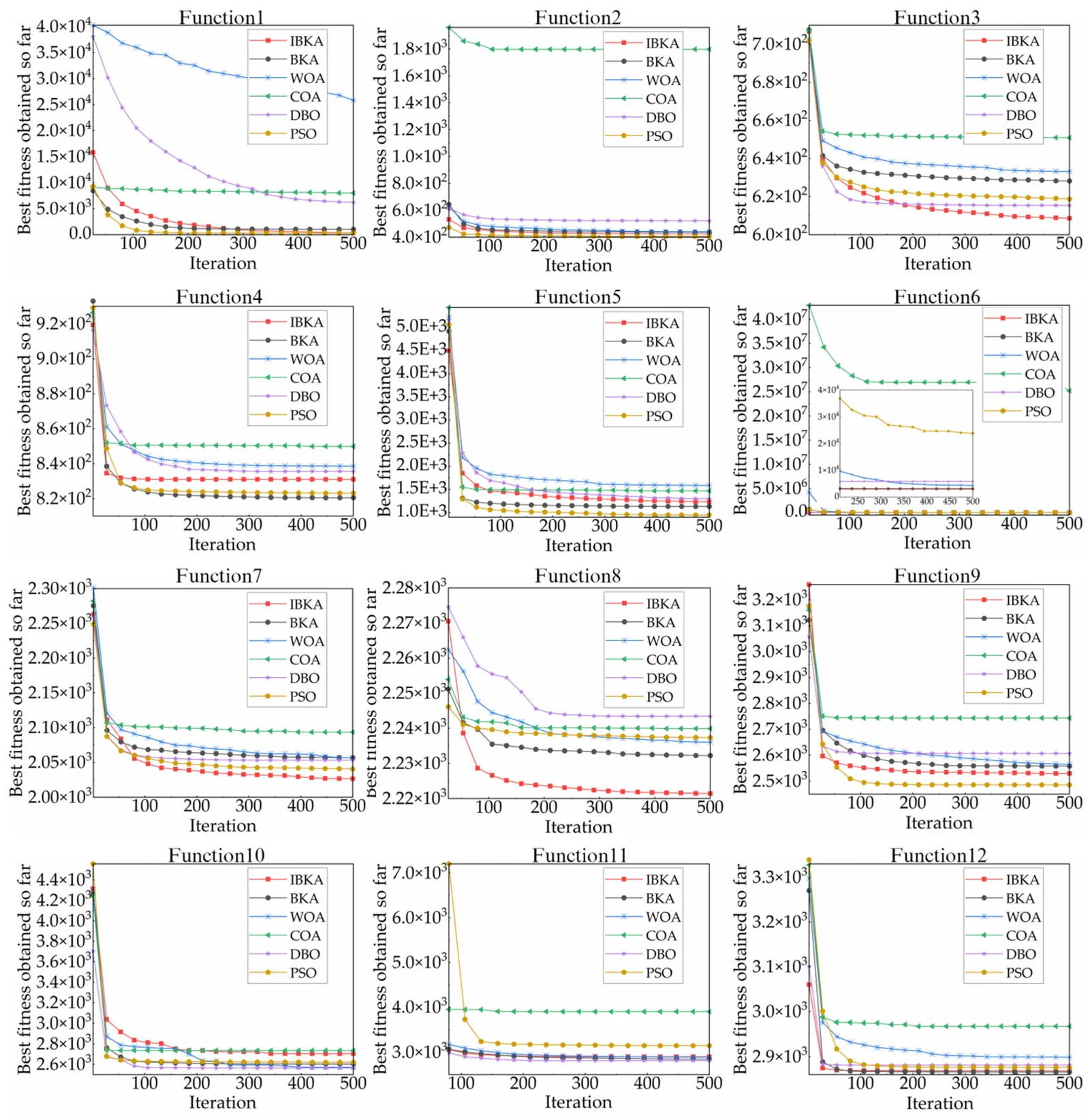
Convergence curves of the test functions.

As illustrated in Figure 3 and Table 4, the results indicate that IBKA achieves the lowest average Friedman rank among all compared algorithms on the CEC2022 benchmark. Compared with the baseline BKA, it exhibits superior performance on F1, F2, F3, F6, F7, F8 and F9; comparable performance on F12; and relatively weaker performance on F4, F5, F10 and F11. IBKA ranks first overall against other competitors, even though it slightly underperforms PSO on F1, F2, F5 and F9 and DBO on F10 and F11. The most remarkable improvements are found in F1 (reduced from 1.04 × 10^3^ to 3.54 × 10^2^) and F6 (decreased from 3.12 × 10^3^ to 2.94 × 10^3^, with a four-order-of-magnitude advantage over COA). These results verify the overall effectiveness and competitive robustness of the proposed improvement strategies.

#### 3.2.4. Complexity Analysis of the Improved Algorithm

After verifying the favorable convergence performance and optimization capability of the IBKA algorithm, this section further evaluates the effect of improvement strategies on algorithm execution efficiency from the perspective of computational complexity. As a critical indicator for assessing optimization execution efficiency, algorithm complexity mainly consists of two dimensions: time complexity and space complexity. The computational complexity of both BKA and IBKA is primarily determined by the population size (pop), search dimension (dim), and maximum number of iterations (T).

Analysis of algorithm logic reveals that the time complexity of BKA is O(T × pop × dim). Although Gaussian chaotic mapping, guidance-pool strategy and adaptive step-size strategy are introduced in IBKA, these improvements do not bring additional nested loops or extra iterative steps. For instance, Gaussian chaotic mapping replaces random initialization while retaining a computational complexity of O(pop × dim). During iterations, the construction of the guidance pool and the calculation of adaptive step sizes only involve linear updates for individuals, without altering the overall time order of the algorithm. Accordingly, IBKA maintains a time complexity of O(T × pop × dim), which is identical in order of magnitude to that of the original BKA. In terms of space complexity, auxiliary variables such as the guidance pool are introduced in IBKA, which leads to a slight increase in memory consumption. Nevertheless, such overhead belongs to low-order terms compared with the storage cost of the population matrix. When tackling high-dimensional optimization problems, the population size (pop) and maximum iterations (T) are generally much larger than the search dimension (dim). Hence, the extra storage overhead caused by the improved strategies does not change the order-of-magnitude of the algorithm’s space complexity.

In summary, IBKA achieves remarkable improvement in optimization performance while maintaining comparable computational complexity to BKA, demonstrating the high-efficiency and low-overhead advantages of the improved algorithm for practical applications.

### 3.3. Hyperparameter Optimization and Unbiased Evaluation Architecture

#### 3.3.1. Hyperparameter Space and Algorithm Configuration

To address the challenge of high-dimensional and strong nonlinearity in dynamic modulus prediction for fiber-reinforced asphalt mixtures, this study adopts IBKA to optimize the hyperparameters of the XGBoost model.

Eight comparative algorithms are introduced to evaluate the optimization performance of the proposed IBKA. For clear comparison logic, these algorithms are divided into three groups: the original algorithm control group (BKA), metaheuristic algorithm group (PSO, WOA, DBO, COA), and conventional tuning group (grid search GS, random search RS, Bayesian optimization BO). The three groups are adopted to verify the effectiveness of IBKA against the original algorithm, mainstream metaheuristic algorithms, and classic hyperparameter-tuning strategies, respectively.

For metaheuristic algorithms, the population size is uniformly set as N = 30, and the maximum number of iterations as T = 100. The fitness function for each algorithm in the inner loop is defined as the root-mean-square error (RMSE) on the validation set. For the conventional baseline algorithms, GS adopts deterministic exhaustive traversal. A discrete coordinate system of 4 × 3 × 3 is established over three major dimensions—maximum depth (3, 6, 9, 12), learning rate (0.01, 0.05, 0.1), and number of trees (100, 350, 600)—to perform full traversal over 36 rigid grid points. RS performs 100 independent random samplings within the hyperparameter space. For BO, a Gaussian-process-based posterior model with the Matérn 5/2 covariance kernel (ν = 2.5) is employed, consisting of 10 initial random samples and 30 sequential iterations using the lower-confidence-bound (LCB) acquisition function. Meanwhile, a unified hyperparameter search space is applied to all models to guarantee fair algorithm comparison. The hyperparameter search space for the XGBoost model is presented in Table 5.

#### 3.3.2. Nested Cross-Validation

To achieve mathematical isolation between hyperparameter optimization and model performance evaluation and avoid optimistic bias during the optimization procedure, this study establishes a methodological framework of nested cross-validation (NCV).

First, the original dataset is split into an 80% training dataset and a 20% independent test set. During hyperparameter optimization and model training, this independent test set is not involved in any model fitting or parameter calibration and is only used for the final evaluation of generalization performance. Afterward, five-fold cross-validation is performed on the 80% training dataset in the outer loop. In each outer iteration, one fold is taken in turn as the outer validation set, and the remaining four folds are combined to form the outer training set. Hyperparameter optimization is entirely implemented inside the outer training set via five-fold cross-validation in the inner loop. Within the inner loop, candidate hyperparameter configurations are evaluated solely based on the outer training set, which ensures that the outer validation set is excluded from hyperparameter selection. This realizes the independence between parameter calibration and performance assessment.

Upon termination of the inner optimization loop and determination of the optimal hyperparameter combination, the model is retrained using the complete current outer training set, and predictions are generated on the corresponding outer validation set to obtain out-of-fold prediction results. The outer-loop evaluation procedure is independently repeated across the five mutually exclusive folds. The unbiased performance estimate of NCV is obtained by averaging the five outer-fold validation metrics.

**Figure 4:**
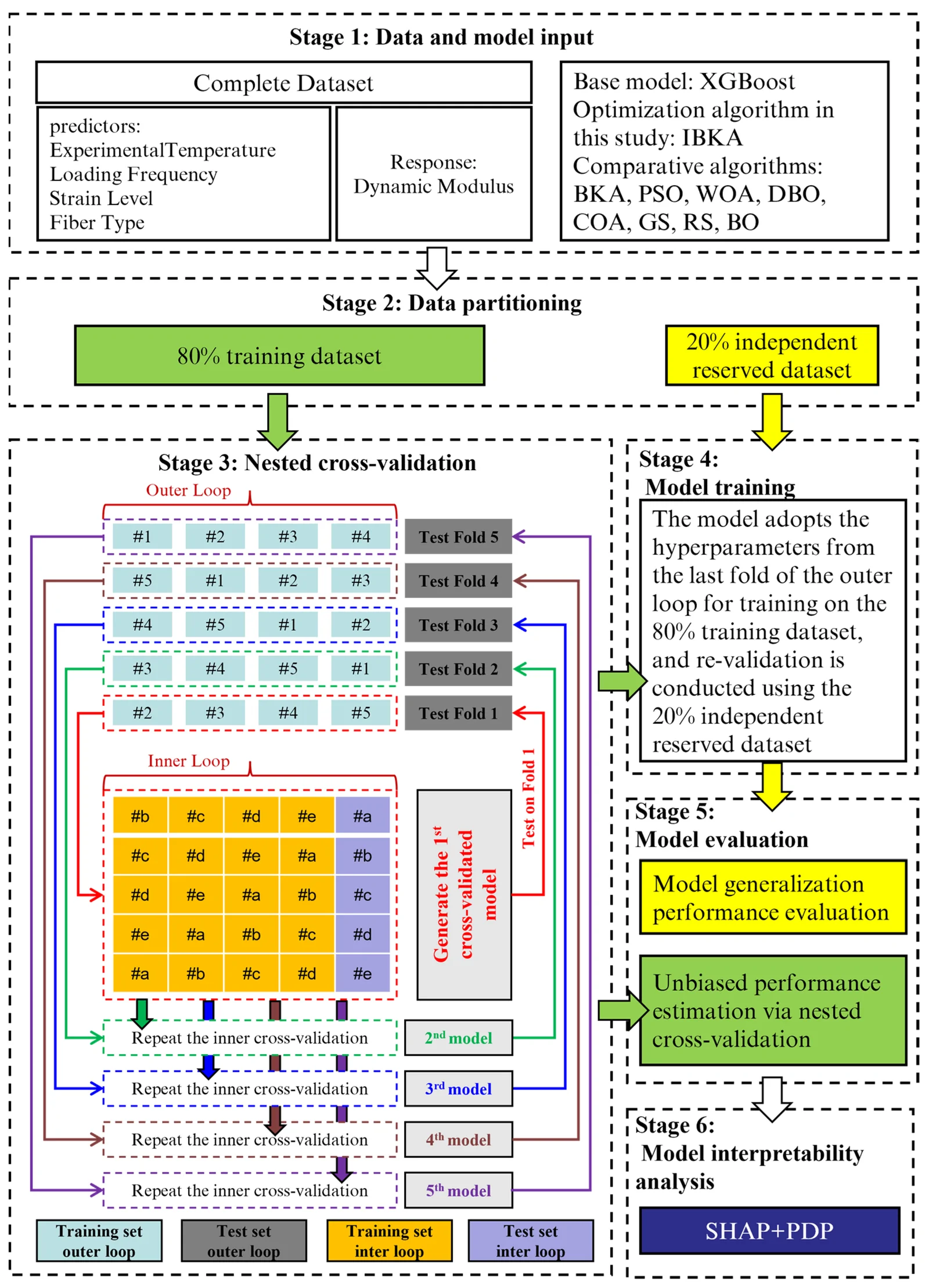
Overall Flowchart.

After the completion of NCV, certain fluctuations exist among the optimal hyperparameter configurations yielded by each outer fold. Therefore, the hyperparameter set determined in the final outer iteration is adopted in this study, and the model is retrained on the full 80% training dataset. The retrained model is then used to predict on the 20% held-out independent test set for evaluating the final generalization performance. The overall workflow of five-fold NCV is illustrated in Figure 4.

### 3.4. Performance Metrics

To comprehensively analyze and evaluate the performance of the DM prediction model, a multi-metric evaluation system is established in this section from three dimensions: prediction accuracy, uncertainty, and comprehensive performance.

For the evaluation of prediction accuracy, mean absolute error (MAE), mean absolute percentage error (MAPE), root-mean-square error (RMSE), mean bias error (MBE), and coefficient of determination (R^2^) are adopted in this study to quantitatively assess the basic fitting accuracy of the model [49,50,51,52]:

(15) $$MAE=\frac{1}{k}\sum_{i=1}^{k}\;|y_{i}-{\hat{y}}_{i}|$$

(16) $$MAPE=\frac{1}{k}\sum_{i=1}^{k}\;\left|\frac{y_{i}-{\hat{y}}_{i}}{y_{i}}\right|$$

(17) $$RMSE=\sqrt{\frac{1}{k}\sum_{i=1}^{k}\;{\left(y_{i}-{\hat{y}}_{i}\right)}^{2}}$$

(18) $$R^{2}=1-\frac{\sum_{i=1}^{k}\;{\left(y_{i}-{\hat{y}}_{i}\right)}^{2}}{\sum_{i=1}^{k}\;{\left(y_{i}-\frac{1}{k}\sum_{i=1}^{k}\;y_{i}\right)}^{2}}$$

(19) $$MBE=\frac{1}{k}\sum_{i=1}^{k}\;\left(y_{i}-{\hat{y}}_{i}\right)$$

For the uncertainty assessment of model predictions, the t-statistic and 95% uncertainty prediction interval (U_95_) are introduced [53]:

(20) $$t-stat=\sqrt{\frac{(k-1){MBE}^{2}}{{RMSE}^{2}-{MBE}^{2}}}$$

(21) $$U_{95}=\sqrt{{1.96(SD}^{2}+{RMSE}^{2})}$$

For comprehensive evaluation of the overall model performance, the performance index (PI) and scatter index (SI) are employed [53]:

(22) $$PI=\frac{1}{{Actual}_{avg}}\frac{RMSE}{\sqrt{R^{2}+1}}$$

(23) $$SI=\frac{RMSE}{{Actual}_{avg}}\times 10$$

### 3.5. Interpretability Analysis of the Prediction Model

To improve the interpretability of the machine-learning model and quantitatively evaluate the influence of each input feature on dynamic modulus prediction, this study introduces the SHapley Additive exPlanations (SHAP) method derived from cooperative game theory. SHAP treats each input feature as an independent contribution factor and regards the final model prediction as the joint effect of all features. Its constructed additive local linear explanation model is expressed as:

(24) $$g(z^{\prime })=\phi_{0}+\sum_{j=1}^{M}\;\phi_{j}z_{j}^{\prime }$$

where $g\left(z^{\prime }\right)$ denotes the local surrogate model; M is the number of input features; $z_{j}^{\prime }\in \{0,1{\}}^{M}$ is a binary vector indicating the presence or absence of features; $\phi_{0}$ is the baseline value (expectation of predicted values over all training samples); and $\phi_{j}$ represents the Shapley attribution value of the j-th feature. For any given input feature i, the mathematical formula for calculating the core Shapley value is defined as follows:

(25) $$\phi_{i}(f)=\sum_{S\subseteq F\setminus \{i\}}\;\frac{|S|!(|F|-|S|-1)!}{|F|!}[f(S\cup \{i\})-f(S)]$$

where F denotes the full set of input features; S is any feature subset excluding feature i; $\left|F\right|$ and $\left|S\right|$ represent the cardinalities (number of elements) of the corresponding sets; $f\left(S\right)$ is the conditional expected prediction output of the model under the feature subset S; and $\left[f\left(S\cup \{i\}\right)-f\left(S\right)\right]$ quantifies the marginal contribution induced by the introduction of feature i.

As a complement, Partial Dependence Plot (PDP) is further introduced to decouple the nonlinear marginal effects of a single feature or two-feature interaction on dynamic modulus. Its mathematical expression for marginal integral averaging is defined as:

(26) $${\hat{f}}_{x_{S}}(x_{S})=\frac{1}{n}\sum_{i=1}^{n}\;f(x_{S},x_{C,i})$$

where $x_{S}$ denotes the target feature for analysis; $x_{C,i}$ represents the complement features other than $x_{S}$ for the i-th sample; and n is the total number of samples.

## 4. Results and Discussion

Based on the previously constructed IBKA–XGBoost dynamic modulus prediction model and the NCV evaluation framework, this section systematically presents and discusses the empirical results. First, the inner validation performances of XGBoost combined with different optimization algorithms are compared via NCV, followed by ablation experiments to quantify the contributions of the three improved mechanisms to the original BKA algorithm. The engineering significance of model robustness is then discussed to clarify the practical value of the prediction accuracy improvement observed in the NCV results. Next, the generalization capability of the optimal model is verified on the independent test set, with permutation significance tests performed to rule out random fitting effects. Finally, SHAP and PDP are adopted for model interpretability analysis to reveal the action mechanism of input features on dynamic modulus.

### 4.1. Result Analysis of NCV

#### 4.1.1. Performance Evaluation and Comparison

**Figure 5:**
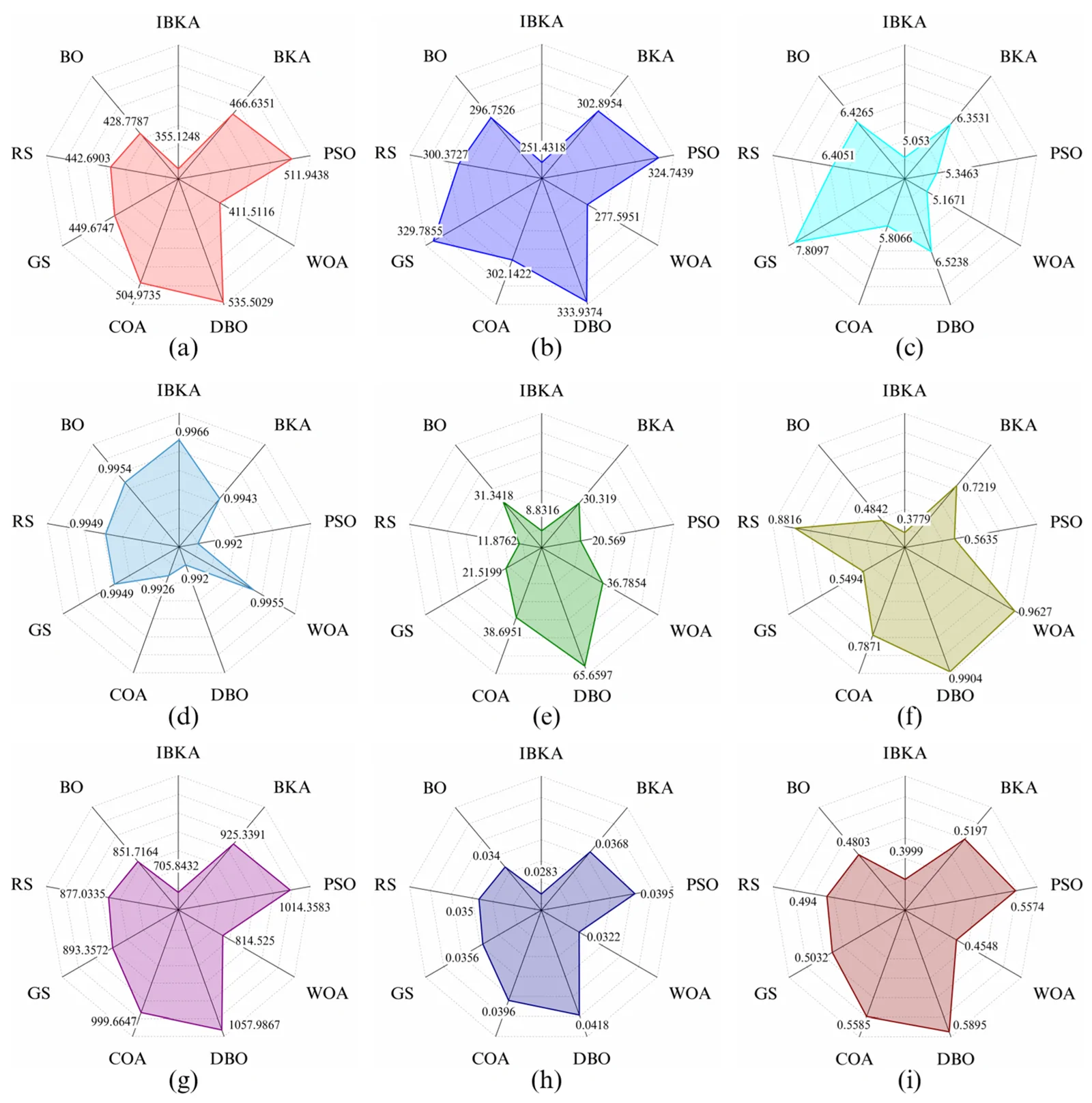
Comparison of average metrics of each model in NCV: (**a**) RMSE; (**b**) MAE; (**c**) MAPE; (**d**) R^2^; (**e**) MBE; (**f**) t-stat; (**g**) U_95_; (**h**) PI; (**i**) SI.

**Figure 6:**
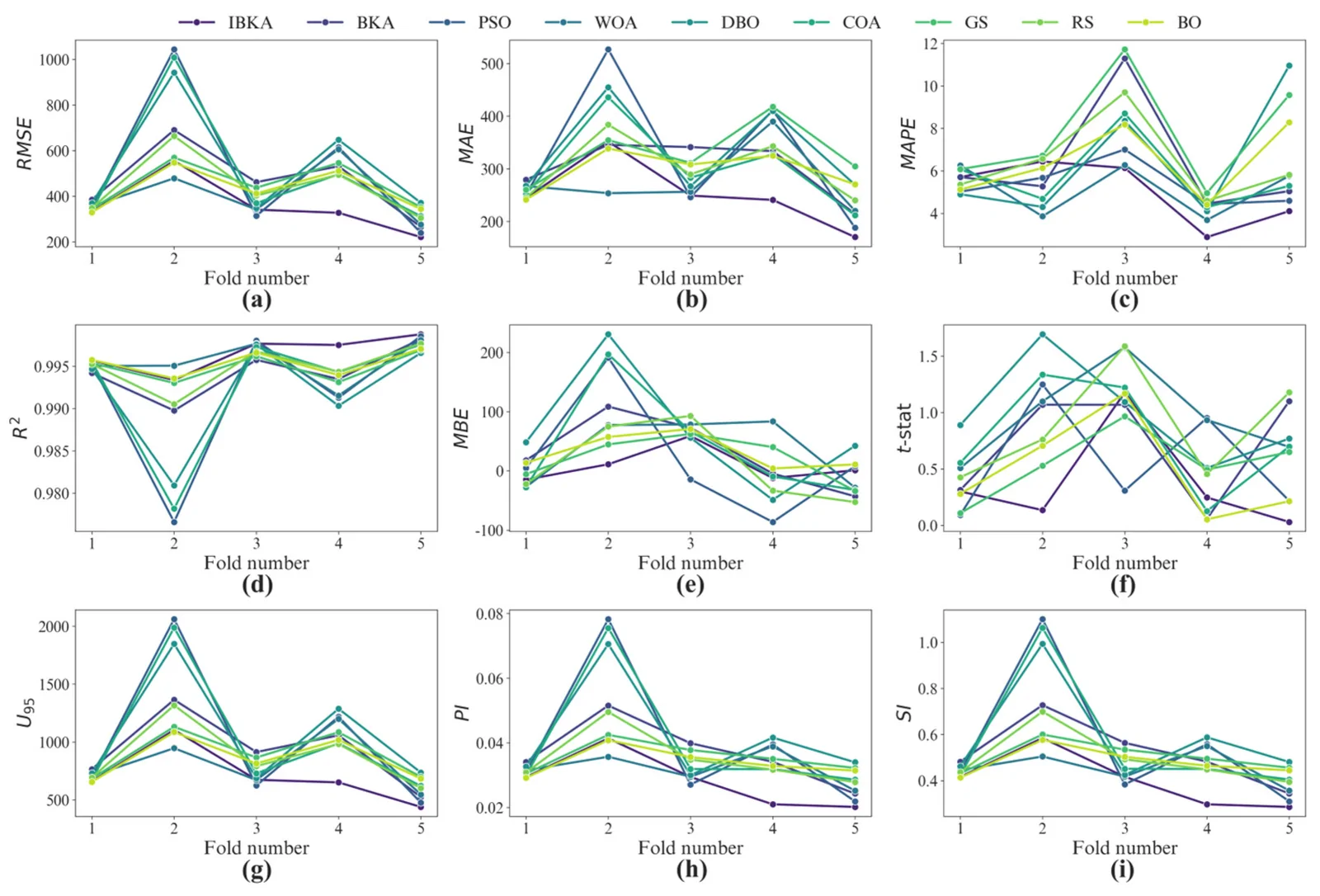
Distribution of metrics across five outer folds in NCV for each model: (**a**) RMSE; (**b**) MAE; (**c**) MAPE; (**d**) R^2^; (**e**) MBE; (**f**) t-stat; (**g**) U_95_; (**h**) PI; (**i**) SI.

In this study, a five-fold NCV framework was adopted to systematically compare and evaluate the performance of XGBoost models integrated with different optimizers. All nine comparative models were categorized into three groups for progressive comparison according to algorithmic principles, including the baseline algorithm group (original BKA–XGBoost), the metaheuristic optimization group (PSO–XGBoost, WOA–XGBoost, DBO–XGBoost, and COA–XGBoost), and the conventional parameter tuning group (GS–XGBoost, RS–XGBoost, and BO–XGBoost). For visual clarity, abbreviated algorithm names are used in Figure 5 and Figure 6, all of which correspond to the XGBoost models optimized by the aforementioned algorithms.

The prediction results of all model groups are illustrated in Figure 5. In terms of prediction accuracy, IBKA–XGBoost achieved the best performance across all five evaluation metrics (RMSE = 355.1248 MPa, MAE = 251.4318MPa, MAPE = 5.053%, R^2^ = 0.9966, MBE = 8.8316 MPa), outperforming BKA–XGBoost, four metaheuristic algorithms (PSO, WOA, DBO, COA), and three conventional tuning strategies (GS, RS, BO). Compared with the baseline BKA–XGBoost, RMSE, MAE, and MAPE decreased by 23.9%, 17.0%, and 20.5%, respectively, while the absolute MBE was reduced by 70.9%. Within the metaheuristic group, WOA–XGBoost ranked second (RMSE = 411.5116 MPa), and IBKA–XGBoost further reduced RMSE by 13.7% relative to it. Among conventional tuning methods, BO–XGBoost performed the best (RMSE = 428.7787 MPa), with IBKA–XGBoost achieving a 17.2% reduction. Regarding predictive uncertainty, IBKA–XGBoost exhibited the narrowest interval (U_95_ = 705.8432 MPa) and a t-statistic closest to zero (0.3779), reducing U_95_ by 23.7% compared with BKA–XGBoost (925.3391 MPa) and by 13.3% and 17.1% relative to WOA–XGBoost and BO–XGBoost, respectively. In terms of comprehensive performance, IBKA–XGBoost achieved the lowest PI (0.0283) and SI (0.3999), decreasing by 23.1% and 23.0% against BKA–XGBoost and by 13.8% and 13.7% against WOA–XGBoost, respectively. These consistent advantages across all three evaluation dimensions and comparison groups confirm that the improved optimization strategy significantly enhances the point estimation accuracy, interval estimation reliability, and overall predictive efficacy of the XGBoost model.

Collectively, the five-fold NCV results demonstrate that the proposed IBKA–XGBoost achieves optimal performance across three core evaluation dimensions, including prediction accuracy, predictive uncertainty, and comprehensive robustness. These findings strongly validate the integrated efficacy of the three improvement strategies in substantially enhancing the overall predictive capability of the XGBoost model.

Figure 6 presents the distribution of evaluation metrics across the five outer folds for each model. IBKA–XGBoost achieved the lowest average RMSE (355.1248 MPa) among the metaheuristic optimization models and maintained a consistently high R^2^ (>0.993) across all folds. Its fold-to-fold RMSE fluctuation (range: 334.0452 MPa, SD: 109.0265 MPa) was comparable to that of WOA (range: 329.6295 MPa, SD: 117.1256 MPa) and markedly lower than that of BKA (range: 424.3298 MPa, SD: 142.2448 MPa), PSO (range: 804.3794 MPa, SD: 294.5871 MPa), DBO (range: 594.8022 MPa, SD: 231.5584 MPa), and COA (range: 695.6232 MPa, SD: 259.4095 MPa). Notably, GS and BO exhibited even smaller internal fluctuations (range: 225.2458 MPa and 217.6089 MPa, SD: 94.1441 MPa and 87.5010 MPa, respectively), yet both showed substantial performance degradation on the independent test set (see Section 4.2.1), with relative increases of 19.7% and 34.4% compared to their NCV results, respectively, which indicates that low internal variability does not necessarily ensure generalization to unseen data. Across all metrics, IBKA–XGBoost demonstrated the most consistent performance, which confirms its robustness against varying data partitions while maintaining strong generalization capability.

#### 4.1.2. Ablation Experiments

To quantitatively evaluate the individual contributions of the three improvement strategies—Gaussian chaotic mapping (S1), guiding pool strategy (S2), and adaptive step-size strategy (S3)—ablation experiments were conducted with the original BKA as the baseline. Four variants (BKA-S1, BKA-S2, BKA-S3, and the full IBKA integrating all three strategies) were tested, with the quantitative results presented in Table 6.

All three individual strategies effectively enhanced the predictive performance of the baseline BKA–XGBoost model. S1 reduced RMSE by 7.6%, attributable to improved initial population uniformity and global search diversity via chaotic mapping. S2 achieved a 72.8% reduction in absolute MBE, benefiting from the guiding pool mechanism that integrates optimal and mean population information to reasonably direct the search. S3 delivered the best overall performance among single strategies, reducing MAPE by 17.4% and yielding the narrowest U_95_ interval, which demonstrates the superiority of adaptive step-size in balancing exploration and exploitation. When synergistically integrated, IBKA–XGBoost achieved an RMSE of 355.1248 MPa (a total reduction of 23.9%, R^2^ = 0.9966), a U_95_ of 705.8432 MPa (a reduction of 23.7%), and PI/SI reductions of 23.1% each—all substantially surpassing the linear superposition of individual gains (21.0%). This significant discrepancy verifies positive interactive effects and synergistic enhancement among the three modules rather than mere functional repetition.

From the perspective of algorithm operational logic, S1 optimizes initial population quality to broaden global search coverage; S2 employs the guiding pool mechanism to prevent premature convergence and correct search deviations; S3 dynamically adjusts iteration step size to adapt to diverse optimization stages. Collectively, the three strategies form a sequential enhancement chain of “initial population coverage → search direction guidance → refined local exploitation”, achieving complementary functions and progressive optimization across different algorithmic phases. The simultaneous declines in PI and SI further confirm that the performance gains in accuracy do not come at the cost of stability but rather realize coordinated improvements in both predictive precision and model robustness.

#### 4.1.3. Engineering Significance of Model Robustness

The NCV results presented above demonstrate that the IBKA–XGBoost model achieves a certain degree of improvement in prediction accuracy over the comparison models. However, a further question warrants discussion: whether the difference in prediction error, approximately 300–400 MPa in RMSE, carries substantive significance in pavement design practice. This can be addressed from two perspectives.

The first regards the propagation effect of prediction error in structural response calculation. Dynamic modulus is a core input parameter for structural response calculation in mechanistic–empirical pavement design methods. It directly affects the accuracy of key mechanical responses, such as tensile strain at the bottom of the asphalt layer and compressive strain at the top of the subgrade, thereby influencing the prediction of fatigue cracking and permanent deformation. Although this study does not directly perform full-section structural calculations, it can be qualitatively stated that deviations in input parameters propagate through the mechanical model to the response quantities. The reasonable range of dynamic modulus for asphalt mixtures spans from thousands to tens of thousands of MPa, and the model’s prediction error of approximately 300–400 MPa lies at the lower end of this magnitude, providing a certain reference value for material performance evaluation at the preliminary design stage.

The second perspective regards robustness as an independent engineering quality. More noteworthy than the improvement in accuracy is the model’s robustness to data partitioning. In engineering practice, experimental data are often derived from a limited number of specimen tests, and the training samples can hardly exhaust all possible combinations of working conditions. In this context, the degree to which model performance depends on the specific training data partition directly determines its reliability on unseen data. The five-fold NCV results (Figure 6) show that the fold-to-fold RMSE range of IBKA–XGBoost is 334.0452 MPa, while the fluctuation ranges of other comparison models are considerably larger: BKA is 424.3298 MPa, PSO is 804.3794 MPa (approximately 2.4 times that of IBKA), and DBO is 594.8022 MPa (approximately 1.8 times that of IBKA). This indicates that under limited sample conditions, IBKA maintains stable performance estimates across different data partitions, without substantial fluctuations due to the specific composition of the training set. For pavement design, a model that provides consistent predictions across different data partitions holds greater engineering practical value than one that performs well only under a specific data partition.

### 4.2. Independent Holdout Set Validation and Statistical Significance Analysis

#### 4.2.1. Generalization Performance Evaluation

**Figure 7:**
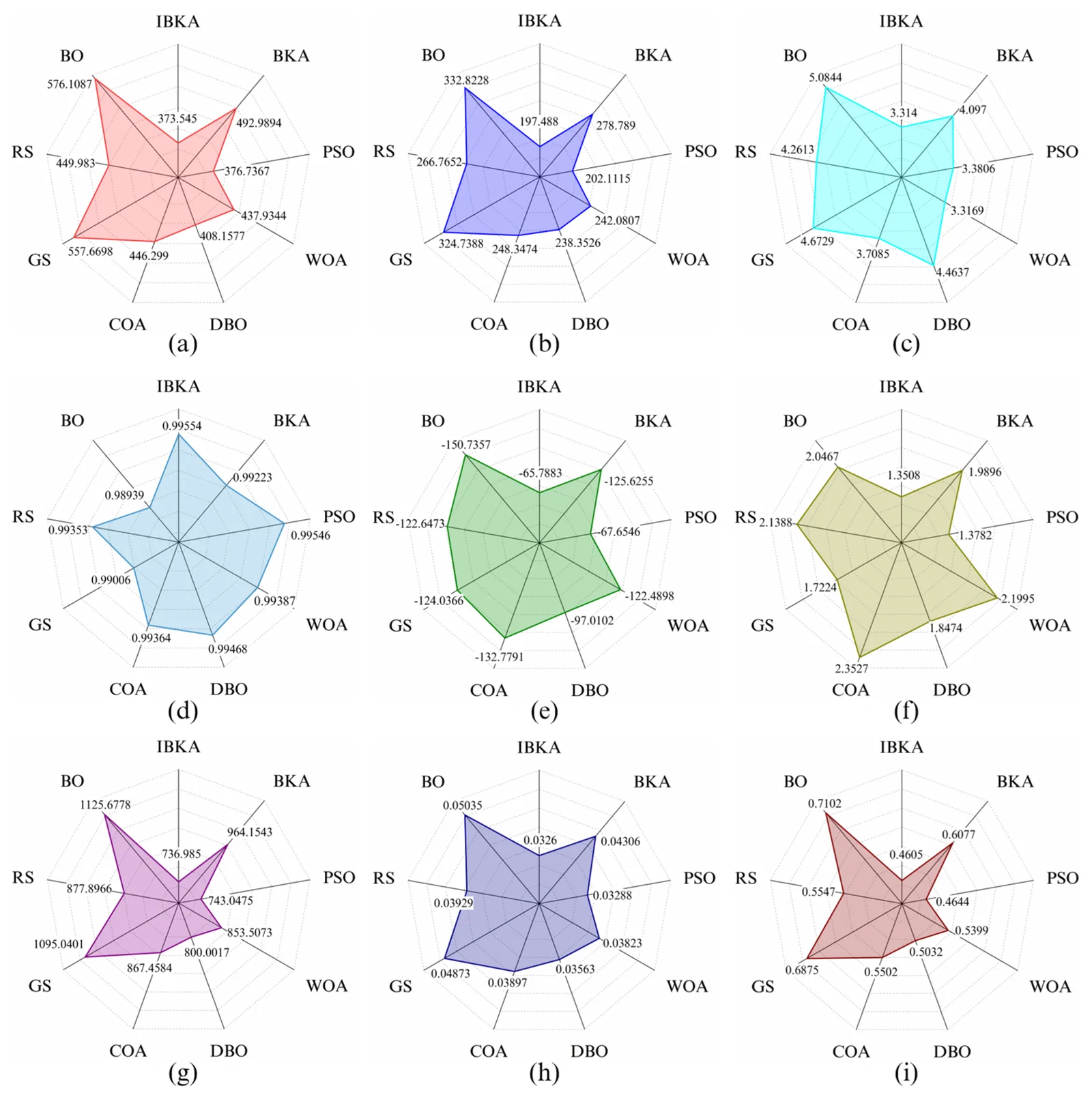
Comparison of metrics of each model on the independent test set: (**a**) RMSE; (**b**) MAE; (**c**) MAPE; (**d**) R^2^; (**e**) MBE; (**f**) t-stat; (**g**) U_95_; (**h**) PI; (**i**) SI.

**Figure 8:**
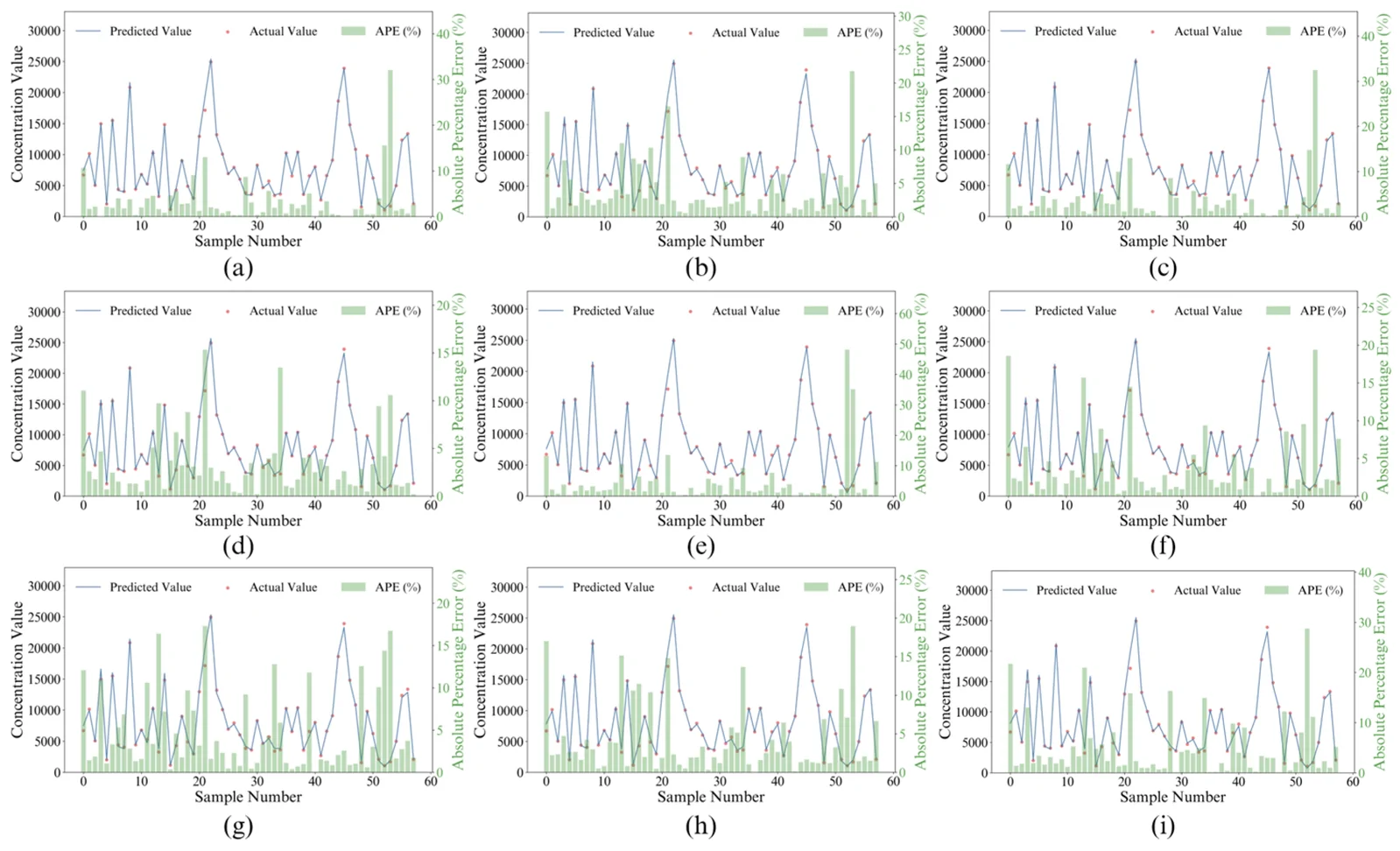
Scatter plots of predicted versus measured values and absolute percentage error distributions on the independent test set for each model: (**a**) IBKA–XGBoost; (**b**) BKA–XGBoost; (**c**) PSO–XGBoost; (**d**) WOA–XGBoost; (**e**) DBO–XGBoost; (**f**) COA–XGBoost; (**g**) GS–XGBoost; (**h**) RS–XGBoost; (**i**) BO–XGBoost.

Although NCV has verified the internal accuracy and stability of the proposed model, practical engineering applications demand reliable performance on fully unseen data. To ensure a strictly unbiased generalization assessment, the optimal hyperparameter configuration for each algorithm was first determined via the inner-loop optimization of NCV—with the final configurations recorded in Table 7—then used to retrain the model on the entire training set (80% of the full dataset). The resulting models were subsequently evaluated once on the reserved 20% independent test set, which had been completely isolated throughout all training and hyperparameter selection procedures. The comprehensive evaluation metrics of all competing models on this external set are summarized in Figure 7, with scatter plots in Figure 8.

On the independent test set, IBKA–XGBoost maintained optimal predictive accuracy (RMSE = 373.5450 MPa, MAE = 197.488 MPa, MAPE = 3.314%, R^2^ = 0.9955, MBE = −65.7883 MPa), outperforming BKA–XGBoost with reductions of 24.2%, 29.2%, and 19.1% in RMSE, MAE, and MAPE, respectively. Within the metaheuristic group, PSO–XGBoost exhibited the closest performance to IBKA–XGBoost (RMSE = 376.7367 MPa, only 0.85% higher), while DBO–, WOA–, and COA–XGBoost yielded RMSE values 9.3%, 17.2%, and 19.5% higher, respectively. Among conventional tuning methods, RS–XGBoost performed the best (RMSE = 449.983 MPa), with IBKA–XGBoost reducing RMSE by 17.0%, 33.0%, and 35.2% relative to RS–, GS–, and BO–XGBoost, respectively. Notably, BO–XGBoost suffered severe generalization degradation: although it performed well in NCV (RMSE = 428.7787 MPa), its performance substantially degraded to 576.1087 MPa on the independent test set, with a relative increase of 34.4%, and its RMSE surged by 34.4% to 576.1087 MPa on the independent set, ranking last—this reveals the inherent deficiency of Bayesian optimization in generalization stability. In terms of uncertainty and comprehensive performance, IBKA–XGBoost again achieved the lowest U_95_ (736.985 MPa), PI (0.0326), and SI (0.4605), with PSO–XGBoost again ranking second (U_95_ = 743.0475 MPa, PI = 0.0329, SI = 0.4644). By contrast, GS–XGBoost and BO–XGBoost exhibited markedly degraded robustness, with U_95_ values approximately 1.5 times that of IBKA–XGBoost (1095.0401 MPa and 1125.6778 MPa) and PI values exceeding 0.048. The scatter plots in Figure 8 further confirm these findings: IBKA–XGBoost (Figure 8a) shows tightly diagonal-distributed points with the vast majority of absolute percentage errors below 10%, while BO–XGBoost (Figure 8i) displays the most discrete distribution.

It is noteworthy that PSO–XGBoost exhibits a notable discrepancy in performance between NCV and external testing: its NCV RMSE is 511.9438 MPa, which improves to 376.7367 MPa on the independent test set. This can be attributed to its high sensitivity to data partitioning: as shown in Figure 6, the RMSE standard deviation of PSO–XGBoost across outer folds was 294.5871 MPa, considerably higher than the 109.0265 MPa of IBKA–XGBoost, indicating that PSO’s optimization results are highly dependent on specific data splits. Benefiting from the synergistic effect of the three improved strategies, IBKA–XGBoost consistently approximates the global optimum even under limited sample conditions—a property of greater engineering significance than marginal improvements in point estimation accuracy, given that training data scales in road engineering are often constrained by experimental costs.

#### 4.2.2. Permutation-Based Significance Test

To eliminate the possibility that the superior model performance originates from accidental random matching between input features and target variables, a Monte Carlo permutation test with 1000 random shuffling iterations was conducted on the independent test set. During each permutation trial, the input feature matrix was fixed while the target variables were randomly shuffled. The RMSE values of IBKA–XGBoost were recalculated after each shuffling to construct the null error distribution under the condition of no genuine feature–target correlation.

**Figure 9:**
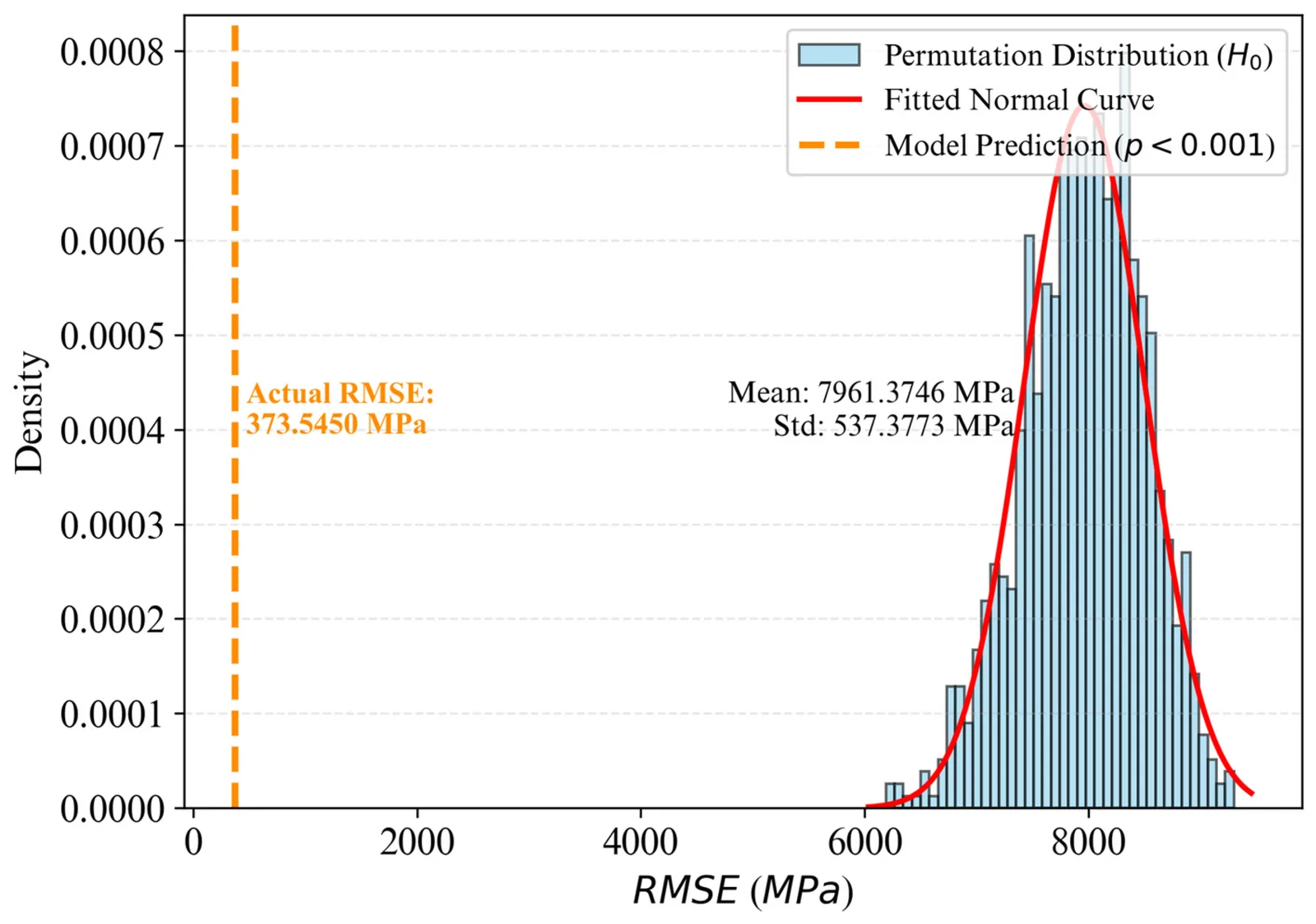
Permutation significance test results for the IBKA–XGBoost model (1000 Monte Carlo simulations).

Figure 9 presents the results of the permutation test. The RMSE values obtained from 1000 permutation iterations follow an approximate normal distribution, with a mean of 7961.3746 MPa and a standard deviation of 537.3773 MPa. By contrast, the actual RMSE of IBKA–XGBoost on the original dataset is 373.5450 MPa, which lies at the far left tail of the null distribution and ranks as the minimum value among all permutation results, yielding a *p*-value of less than 0.001. This solid statistical evidence demonstrates that the mapping relationship established between input features and dynamic modulus by IBKA–XGBoost reflects genuine mechanical causality rather than accidental data correlation or random overfitting. Accordingly, the inherent associations between the input variables (test temperature, test frequency, strain level, and fiber type) and the output dynamic modulus captured by the proposed model are statistically reliable. This valid foundation further guarantees the scientific validity and credibility of the subsequent SHAP and PDP interpretability analysis.

### 4.3. SHapley Additive exPlanations (SHAP) for Variable Analysis

SHAP analysis was performed on the IBKA–XGBoost model to quantify each feature’s marginal contribution to dynamic modulus prediction and to identify directional correlations between feature values and model outputs, with detailed methodology presented in Section 3.5.

**Figure 10:**
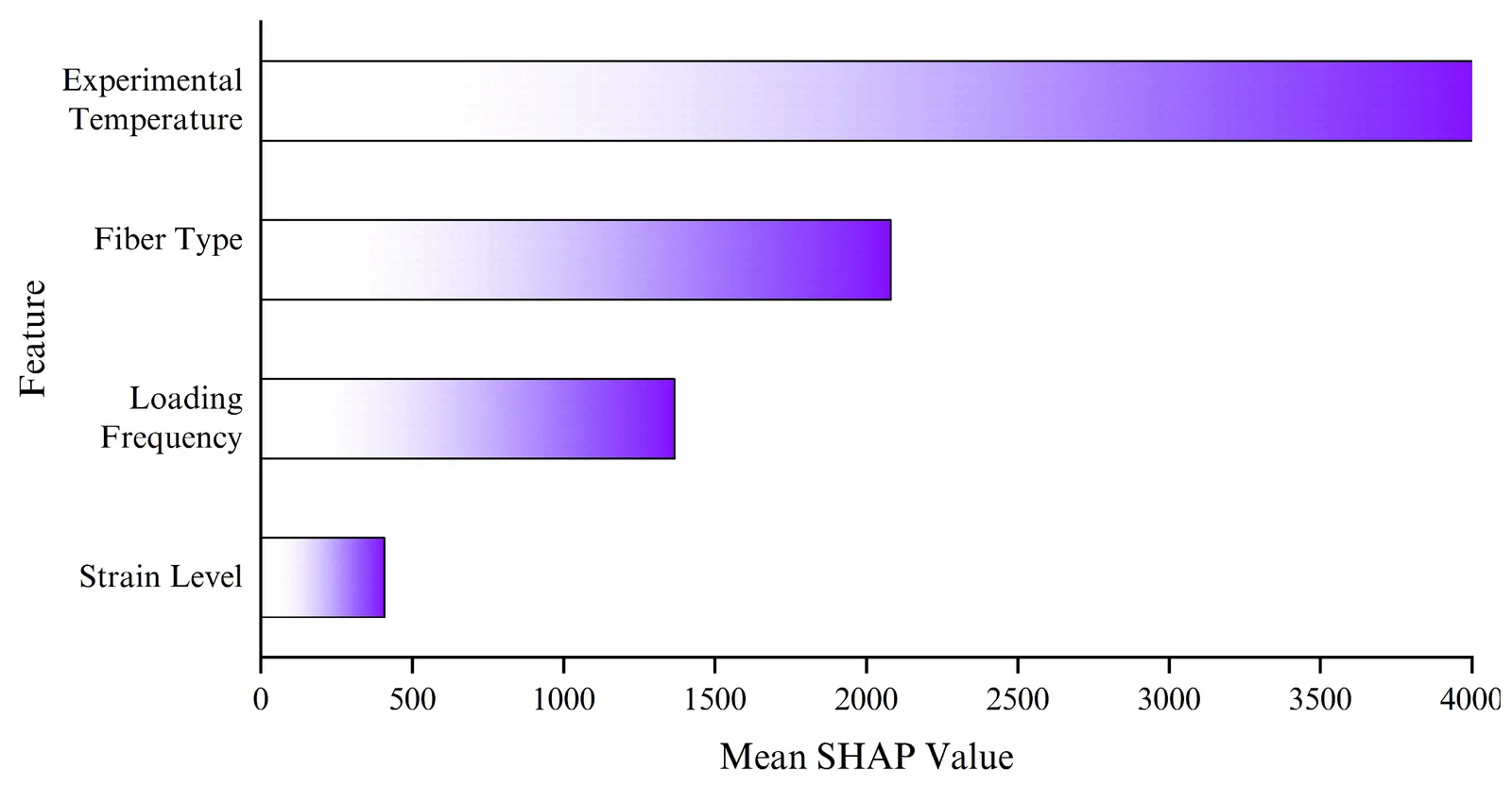
Mean SHAP values of each input feature (global feature importance).

In terms of global feature importance (Figure 10), test temperature ranks first with a contribution proportion of approximately 52.5%, substantially higher than other features, which confirms that temperature dominates the variation in dynamic modulus—consistent with the viscoelastic theory of asphalt mixtures. Fiber type ranks second (26.8%), demonstrating a non-negligible regulatory effect, followed by loading frequency (15.4%), which reflects the core role of time–temperature superposition. Strain level presents the weakest influence (5.4%), which validates the rationality of conducting DMA tests within the linear viscoelastic range of 30–90 με.

**Figure 11:**
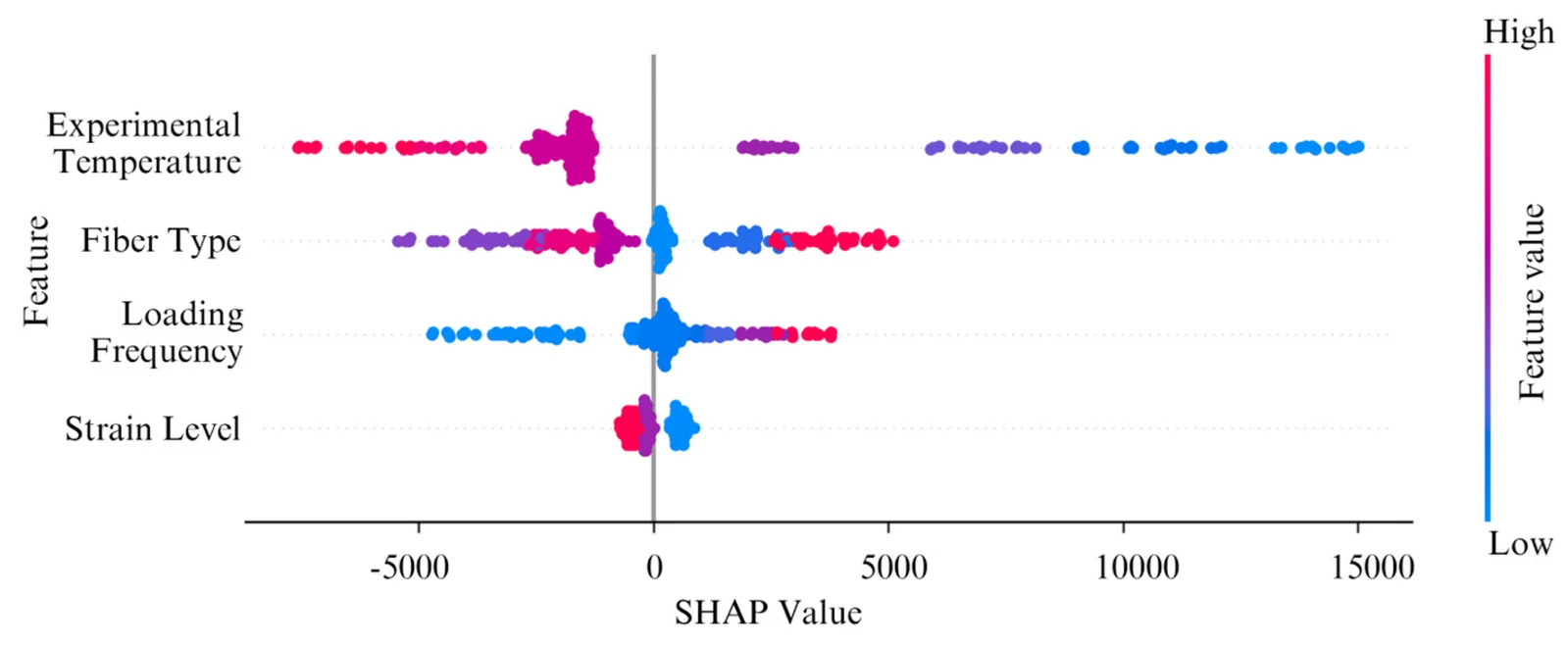
SHAP summary plot of each feature (influence of feature values on prediction results).

The SHAP swarm plot (Figure 11) further reveals directional correlations. For temperature, the SHAP values shift from positive to negative as temperature rises, with negative amplitudes increasing sharply above 20 °C—corresponding to the modulus drop near the glass transition. For frequency, the SHAP values remain positive across the entire range but saturate at high frequencies (>10 Hz), consistent with time–temperature superposition expectations. For fiber type, a clear three-group differentiation emerges: PET1 and CEL form a positive-effect cluster; PET2, PVA, and PAN form a negative-effect cluster; and the control group lies in between. This directional classification provides a clear basis for the subsequent PDP-based physical mechanism interpretation.

### 4.4. Partial Dependence Plot (PDP) Analysis for Explanatory Variables

**Figure 12:**
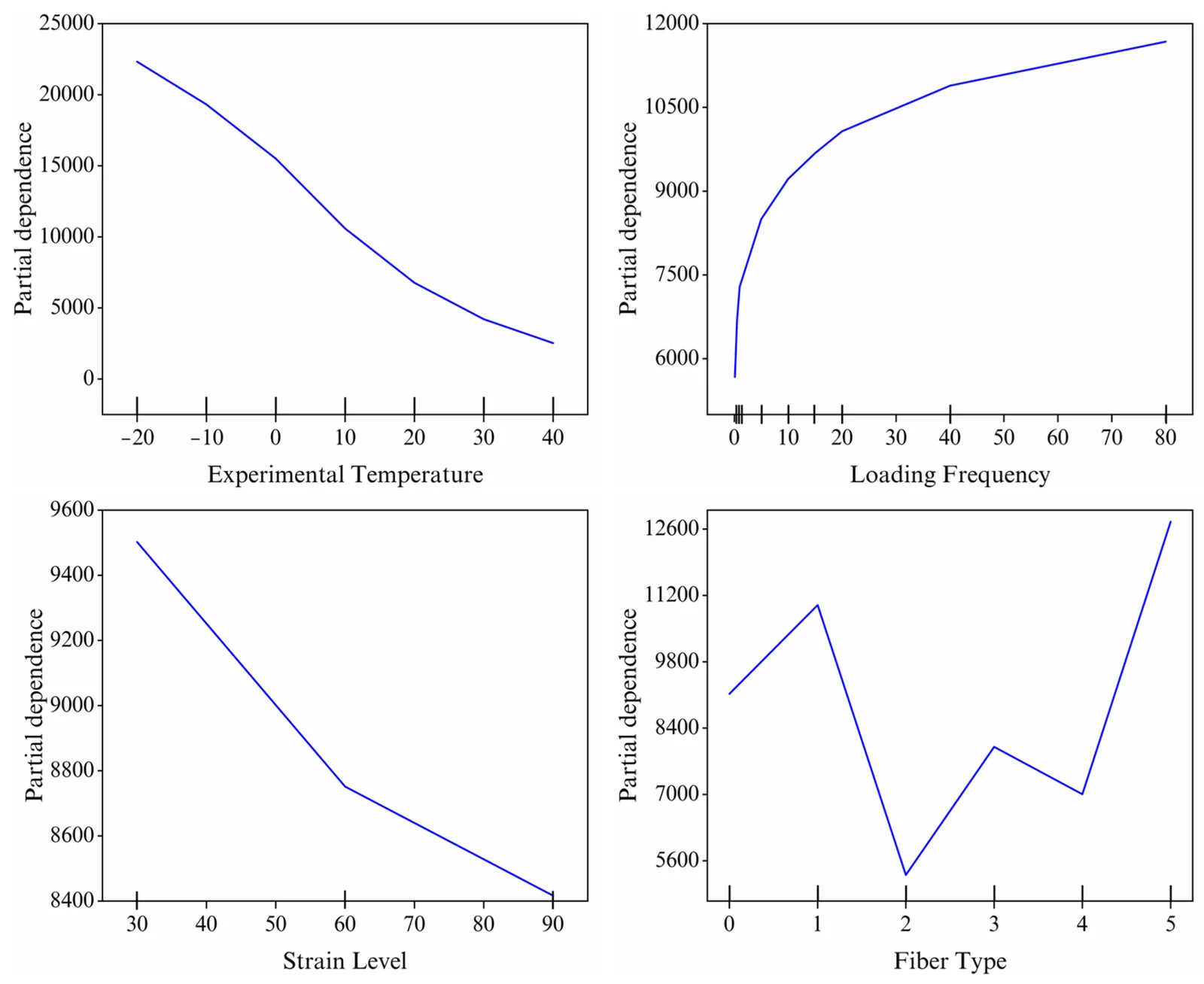
Single-feature PDP.

**Figure 13:**
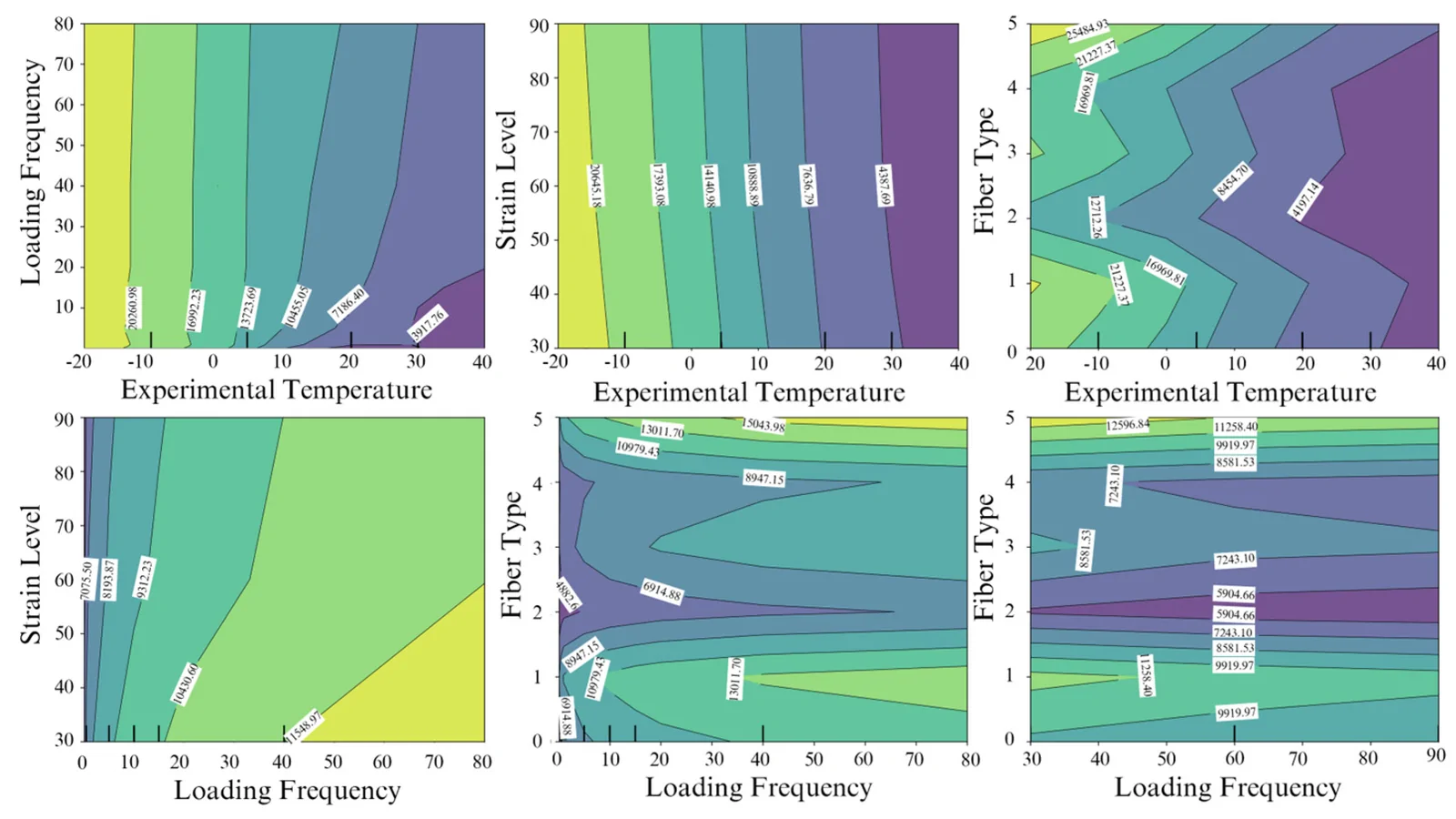
Two-feature interaction PDP.

SHAP analysis provides global importance ranking and directional contributions but cannot reveal nonlinear marginal variations across feature intervals or interactive effects between variables. PDP analysis was therefore adopted for supplementary interpretation, with the results presented in Figure 12 and Figure 13.

The univariate PDP results (Figure 12) indicate that temperature exhibits a monotonic negative correlation with dynamic modulus, with an 88.7% reduction from −20 °C to 40 °C and the most drastic attenuation occurring between −10 °C and 20 °C—corresponding to the glass-to-rubber transition region of asphalt binder. Frequency shows a monotonic positive correlation that gradually saturates at high frequencies, consistent with time–temperature superposition. Strain level presents only a weak negative correlation (overall reduction of 11.4%) within the 30–90 με range, well below the linear viscoelastic threshold, which further validates the rationality of the DMA test design.

For fiber type, the univariate PDP marginal effect ranking (CEL > PET1 > Control > PAN > PVA > PET2) aligns with the SHAP three-group classification. The underlying modification mechanisms are governed by four synergistic physical properties: modulus matching with the asphalt matrix, breaking elongation, interfacial compatibility, and thermal shrinkage behavior. Positive-effect fibers (CEL and PET1) enhance dynamic modulus through two distinct pathways. PET1 exhibits the highest breaking elongation (18.3%) and dry heat shrinkage rate (9.5%) among all fibers. Its high elongation enables strain energy dissipation and stress concentration relief, while the thermal shrinkage generates moderate prestress that tightens interfacial mechanical interlocking; its moderate modulus avoids extreme mismatch with the matrix. CEL, as a flocculent short fiber (length 1.1 mm), relies on its porous structure to adsorb light asphalt components, thickening the mortar and optimizing its spatial distribution, thereby indirectly improving viscoelastic performance. By contrast, negative-effect fibers (PET2, PVA, and PAN) deteriorate modulus through distinct deficiencies. PET2 suffers from insufficient elongation (5.55%, less than one-third of PET1) and low thermal shrinkage (8.5%), which limit both energy dissipation and interfacial compaction. PVA combines ultra-high modulus (39.71 GPa) with strong hydrophilicity (rich in –OH groups), causing severe mechanical mismatch and poor wetting with hydrophobic asphalt—a dual negative effect. PAN, despite adequate strength and moderate elongation (15.6%), has ultra-fine fineness (1.2 dtex) and a hydrophilic surface (moisture content < 2%), which make it prone to pull-out rather than effective stress transfer under weak interfacial bonding.

The bivariate PDP (Figure 13) further reveals pairwise interactions. The temperature–frequency interaction is most prominent: the frequency enhancement is considerably stronger in the low-temperature range (−20 °C to 0 °C) than in the high-temperature range (20 °C to 40 °C), as low temperatures “freeze” molecular chain motion, making the system sensitive to equivalent temperature changes from frequency variation, whereas high-temperature viscous dissipation buffers frequency effects. The temperature–fiber interaction shows that PET1 and CEL exhibit more moderate modulus decay with increasing temperature compared with negative-effect fibers, which indicates that good interfacial compatibility not only enhances absolute modulus but also stabilizes performance across the full temperature range. The frequency–fiber interaction reveals that the ranking of fiber effects remains stable across frequencies, but under low-frequency conditions (<1 Hz), negative-effect fibers show significantly greater modulus attenuation than positive-effect fibers—a critical finding for low-speed, heavy-load road sections such as long longitudinal slopes and toll stations, where PET1 or CEL should be prioritized and PET2 strictly avoided. The frequency–strain interaction indicates that strain’s weakening effect is slightly amplified at low frequencies due to sufficient relaxation time for viscous flow.

In summary, SHAP provides global importance rankings and directional judgment, while PDP further decouples nonlinear marginal effects, interprets fiber physical mechanisms, and reveals pairwise interactions. Cross-validation between the two approaches enhances conclusion reliability. The temperature–frequency joint effect captured by IBKA–XGBoost is consistent with classical time–temperature superposition, and the regulatory role of fiber interfacial compatibility on temperature-dependent performance agrees with fundamental expectations from composite mesomechanics.

## 5. Conclusions

In this study, an XGBoost prediction model for the dynamic modulus of fiber-reinforced asphalt mixtures was established based on the IBKA. The model performance was systematically validated via NCV and independent test set verification. With the combination of SHAP and PDP interpretability analysis, the influence laws of input features and their complex interaction mechanisms were comprehensively revealed, which verifies the effectiveness and reliability of the proposed model for engineering application. The main conclusions are summarized as follows:

1. Benefiting from the triple improvements of Gaussian chaotic mapping, guidance pool strategy, and adaptive step-size strategy, the proposed IBKA effectively addresses the core drawbacks of the original BKA, including uneven population initialization, premature convergence, and fixed step-size adjustment. The improved algorithm ranks first in the CEC2022 benchmark function tests, which thoroughly verifies the feasibility and superiority of the integrated improvement strategies.
2. The IBKA–XGBoost model achieves superior prediction accuracy and generalization performance in both NCV internal verification and independent test set validation. It exhibits the smallest fold-to-fold RMSE fluctuation among the metaheuristic optimization models in five-fold NCV, demonstrating excellent robustness against data partitioning. Furthermore, the permutation test results (*p* < 0.001) exclude the possibility of random fitting. The above findings confirm that the proposed model can simultaneously achieve high prediction accuracy and reliable stability under limited sample data conditions.
3. SHAP and PDP interpretability analyses reveal that temperature serves as the dominant factor affecting the dynamic modulus, with a feature contribution of 52.5%, followed by fiber type (26.8%), loading frequency (15.4%), and strain amplitude (5.4%). The marginal contribution of strain further verifies that the applied strain range of 30–90 με is strictly confined within the linear viscoelastic region, which confirms the reliability of the experimental dataset. The fiber modification effect is synergistically governed by modulus matching degree, breaking elongation, interfacial compatibility, and thermal shrinkage characteristics.
4. In terms of engineering fiber selection, the priority order for conventional road sections is recommended as CEL ≥ PET1 > fiber-free > PAN > PVA > PET2. For low-frequency (<1 Hz) and heavy-load road sections, such as long longitudinal slopes and toll stations, PET1 and CEL are the preferred choices, while PET2 should be strictly avoided. Overall, positive-effect fibers applicable to asphalt mixtures are characterized by a moderate elastic modulus, breaking elongation higher than 15%, a dry heat shrinkage rate of 8–10%, and favorable wettability with asphalt binders.

It is noteworthy that the present conclusions are drawn based on laboratory DMA tests adopting the SAC-13 gradation, five types of fibers, and a single fiber dosage of 0.3%. The applicability of the findings to broader material combinations and complex field service conditions still requires further validation. Future studies will focus on the establishment of a multi-type fiber performance database, the development of transfer learning strategies for asphalt mixture prediction tasks, and cross-scale investigations of the microscale interfacial behavior between fiber and asphalt binder.

## Figures and Tables

**Table 1:** Gap-graded SAC-13 aggregate gradation designed via the power-function method.

**SAC-13 Gradation**	**Passing Rate at Each Sieve Size/%**									
	16	13.2	9.5	4.75	2.36	1.18	0.6	0.3	0.15	0.075
	100	95	62.6	26	20.8	16.7	13.5	10.9	8.7	7

**Table 2:** Comparison of physical and mechanical properties of five types of fibers.

Fiber Type	Tensile Strength/Tensile Force	Modulus/GPa	Breaking Elongation/%	Dry Heat Shrinkage Rate/%	Density/(g·cm^−3^)	Fineness/dtex
PET1	8.66 N	—	18.3	9.5	1.38	156
PET2	>500 MPa	—	5.55	8.5	1.36	156
PAN	>800 MPa	—	15.6	—	1.18	1.2
PVA	1725.28 MPa	39.71	6.47	—	1.291	2.01
CEL	—	—	—	—	1.54	—

**Table 3:** Descriptive Statistical Results of the DM Dataset.

Index	Mean	Std. Dev.	Min	Max	CV/%
Experimental temperature/°C	15.6	14.2	−20.0	40.0	90.6
Loading frequency/Hz	15.1	19.1	0.1	80.0	126.3
Strain level/με	60.1	24.6	30.0	90.0	40.9
Fiber type/Code	—	—	—	—	—
Dynamic modulus/MPa	8726.8	6379.3	603.7	28,831.4	73.1

**Table 4:** Test results of IBKA and comparison algorithms on CEC2022 test suite.

Function	Index	IBKA	BKA	COA	DBO	WOA	PSO
F1	Mean	3.54 × 10^2^	1.04 × 10^3^	8.00 × 10^3^	6.20 × 10^3^	2.58 × 10^4^	**3.11 × 10^2^**
	Std	7.59 × 10^1^	2.77 × 10^3^	1.61 × 10^3^	8.96 × 10^3^	1.40 × 10^4^	**3.38 × 10^0^**
F2	Mean	4.24 × 10^2^	4.37 × 10^2^	1.80 × 10^3^	5.22 × 10^2^	4.40 × 10^2^	**4.05 × 10^2^**
	Std	3.04 × 10^1^	7.27 × 10^1^	6.36 × 10^2^	2.68 × 10^2^	6.77 × 10^1^	**1.19 × 10^1^**
F3	Mean	**6.09 × 10^2^**	6.28 × 10^2^	6.51 × 10^2^	6.15 × 10^2^	6.33 × 10^2^	6.19 × 10^2^
	Std	1.46 × 10^1^	1.14 × 10^1^	**9.68 × 10^0^**	1.18 × 10^1^	1.35 × 10^1^	1.14 × 10^1^
F4	Mean	8.31 × 10^2^	**8.20 × 10^2^**	8.50 × 10^2^	8.36 × 10^2^	8.39 × 10^2^	8.23 × 10^2^
	Std	1.37 × 10^1^	9.08 × 10^0^	8.79 × 10^0^	1.42 × 10^1^	1.80 × 10^1^	**7.87 × 10^0^**
F5	Mean	1.23 × 10^3^	1.13 × 10^3^	1.47 × 10^3^	1.30 × 10^3^	1.59 × 10^3^	**9.46 × 10^2^**
	Std	3.22 × 10^2^	**1.49 × 10^2^**	1.68 × 10^2^	4.19 × 10^2^	4.10 × 10^2^	1.73 × 10^2^
F6	Mean	**2.94 × 10^3^**	3.12 × 10^3^	2.52 × 10^7^	5.82 × 10^3^	4.29 × 10^3^	2.38 × 10^4^
	Std	**1.47 × 10^3^**	2.01 × 10^3^	4.01 × 10^7^	2.12 × 10^3^	2.01 × 10^3^	1.07 × 10^4^
F7	Mean	**2.03 × 10^3^**	2.06 × 10^3^	2.09 × 10^3^	2.05 × 10^3^	2.06 × 10^3^	2.04 × 10^3^
	Std	**1.01 × 10^1^**	3.01 × 10^1^	1.64 × 10^1^	4.10 × 10^1^	2.64 × 10^1^	1.70 × 10^1^
F8	Mean	**2.22 × 10^3^**	2.23 × 10^3^	2.24 × 10^3^	2.24 × 10^3^	2.24 × 10^3^	2.24 × 10^3^
	Std	**2.02 × 10^0^**	2.16 × 10^1^	1.05 × 10^1^	4.31 × 10^1^	7.33 × 10^0^	3.12 × 10^1^
F9	Mean	2.53 × 10^3^	2.56 × 10^3^	2.74 × 10^3^	2.61 × 10^3^	2.56 × 10^3^	**2.49 × 10^3^**
	Std	**1.53 × 10^2^**	6.50 × 10^1^	4.30 × 10^1^	8.43 × 10^1^	3.57 × 10^1^	5.60 × 10^−2^
F10	Mean	2.70 × 10^3^	2.60 × 10^3^	2.74 × 10^3^	**2.56 × 10^3^**	2.57 × 10^3^	2.62 × 10^3^
	Std	2.49 × 10^2^	2.02 × 10^2^	1.35 × 10^2^	**8.55 × 10^1^**	1.44 × 10^2^	1.67 × 10^2^
F11	Mean	2.90 × 10^3^	2.84 × 10^3^	3.90 × 10^3^	**2.81 × 10^3^**	2.89 × 10^3^	3.15 × 10^3^
	Std	**3.73 × 10^−2^**	3.98 × 10^2^	4.02 × 10^2^	1.83 × 10^2^	7.94 × 10^1^	6.32 × 10^2^
F12	Mean	2.87 × 10^3^	**2.87 × 10^3^**	2.97 × 10^3^	2.88 × 10^3^	2.90 × 10^3^	2.88 × 10^3^
	Std	5.51 × 10^0^	**4.98 × 10^0^**	5.17 × 10^1^	1.87 × 10^1^	5.67 × 10^0^	6.04 × 10^1^
Friedman	average ranking	**2.2500**	2.5833	5.7500	3.5833	4.1667	2.6667
Final Ranking		**1**	2	6	4	5	3

Note: Bold values indicate the best performance among all algorithms for each metric.

**Table 5:** Hyperparameter Search Space.

Hyperparameter	Range Explored
Optimal population size	10–100
Maximum tree depth	3–12
Learning rate	0.01–0.1
Number of estimators	100–600
Subsample ratio	0.5–1.0
Column sample by tree	0.5–1.0
L1 regularization	0–0.5
L2 regularization	0–1
Minimum child weight	1–10
Gamma	0–0.1

**Table 6:** Comparison of evaluation metrics for each model in ablation study.

Models	RMSE	MAE	MAPE	R^2^	MBE	t-stat	U_95_	PI	SI
BKA	466.6351	302.8954	6.3531	0.9943	30.319	0.7219	925.3391	0.0368	0.5197
BKA-S1	430.9399	292.4408	5.7047	0.9952	13.2047	0.7746	854.6788	0.0339	0.4791
BKA-S2	439.6334	297.9907	5.4045	0.9949	−8.2321	0.8686	871.0066	0.0343	0.4851
BKA-S3	433.0786	291.2929	5.2508	0.9950	9.2757	0.5907	860.0366	0.0337	0.4766
IBKA	355.1248	251.4318	5.0530	0.9966	8.8316	0.3779	705.8432	0.0283	0.3999

Note: IBKA and BKA denote their corresponding XGBoost models optimized by the respective algorithms. S1, S2, and S3 represent the Gaussian chaotic mapping, guiding pool strategy, and adaptive step-size strategy, respectively. All numerical results correspond to the mean values derived from the five-fold NCV tests.

**Table 7:** Optimized Hyperparameters.

Hyperparameter	Optimal Value								
	IBKA	BKA	PSO	WOA	DBO	COA	GS	RS	BO
Optimal population size	5	6	5	4	11	5	9	5	5
Maximum tree depth	0.0933	0.0585	0.1	0.0817	0.098	0.0944	0.1	0.0437	0.0807
Learning rate	592	572	593	406	385	247	600	541	267
Number of estimators	1	0.6854	0.9237	0.9493	0.922	0.7798	0.75	0.7861	0.7053
Subsample ratio	0.5018	0.8774	0.6944	0.9171	0.6267	0.8146	0.75	0.7897	0.9637
Column sample by tree	8.05 × 10^−10^	0.1550	0	0.0013	0.2490	0.3731	0.25	0.4535	0.0413
L1 regularization	0.357	0.5746	0.6382	0.4615	0.3541	0.6146	0.5	0.8704	0.6770
L2 regularization	1	2	1	1	4	1	6	1	3
Minimum child weight	0.0003	0.0278	0.1	0.099	0.0861	0.0664	0.05	0.0934	0.0265

## Data Availability

The original contributions presented in this study are included in the article. Further inquiries can be directed to the corresponding author.

## Abbreviations

The following abbreviations are used in this manuscript:

DM	Dynamic modulus
DMA	Dynamic mechanical analysis
PET1	Polyethylene terephthalate fiber
PET2	Polyester fiber
PAN	Polyacrylonitrile fiber
PVA	Polyvinyl alcohol fiber
CEL	Lignin fiber
XGBoost	eXtreme Gradient Boosting
GS–XGBoost	Grid search optimization eXtreme Gradient Boosting
RS–XGBoost	Random search optimization eXtreme Gradient Boosting
BO–XGBoost	Bayesian optimization eXtreme Gradient Boosting
PSO–XGBoost	Particle Swarm Optimization eXtreme Gradient Boosting
WOA–XGBoost	Whale Optimization Algorithm optimization eXtreme Gradient Boosting
DBO–XGBoost	Dung Beetle Optimizer optimization eXtreme Gradient Boosting
COA–XGBoost	Coati Optimization Algorithm optimization eXtreme Gradient Boosting
BKA–XGBoost	Black-winged kite algorithm optimization eXtreme Gradient Boosting
IBKA–XGBoost	Improved black-winged kite algorithm optimization eXtreme Gradient Boosting
SHAP	SHapley Additive exPlanations
PDP	Partial Dependence Plot
